# Single-cell transcriptomics identifies a p21-activated kinase important for survival of the zoonotic parasite *Fasciola hepatica*

**DOI:** 10.1016/j.isci.2026.116778

**Published:** 2026-07-16

**Authors:** Oliver Puckelwaldt, Svenja Gramberg, Sagar Ajmera, Janine Koepke, Jamal Shamsara, Christos Samakovlis, Peter Kolb, Simone Haeberlein

**Affiliations:** 1 Institute of Parasitology, Justus Liebig University Giessen, Giessen, Germany; 2 Cardio-Pulmonary Institute (CPI), Giessen, Germany; 3 Department of Pharmaceutical Chemistry, Philipps-Universität Marburg, Marburg, Germany; 4 Core Facility Chemoinformatics and Molecular AI, Philipps-Universität Marburg, Marburg, Germany

**Keywords:** single-cell transcriptomics, scRNASeq, flatworms, liver flukes, *Fasciola hepatica*, drug target, p21-activated kinase, TRPM, stem cells

## Abstract

Knowledge on the cell types and cell-specific gene expression of multicellular pathogens facilitates drug discovery and allows gaining a deeper understanding of pathogen biology. By utilizing single-cell RNA sequencing (scRNA-seq), we analyzed 19,581 cells of a globally prevalent parasitic flatworm, the liver fluke *Fasciola hepatica*, which affects health of both humans and animals. We identified 15 distinct clusters, including stem cells, gonadal, muscle and intestinal cells. Differentiation lineages within this parasite were identified and characterized by integrating RNA velocity and spatial transcriptomics data. Furthermore, an ELF5- and TRPM_PZQ_-expressing cell cluster was discovered, characterized by high expression of protein kinases, including the p21-activated kinase PAK4. Treatment with a PAK4 inhibitor efficiently killed the parasites. These data provide insight into the cellular composition of a complex multicellular pathogen and demonstrate how gene expression at single-cell resolution can serve as a resource for the identification of new drug targets.

## Highlights

• Achieved and validated a cell type dataset for the liver fluke *Fasciola hepatica*
• Spatially resolved the distribution of cell clusters with spatial transcriptomics
• Predicted a lineage of vitelline cells with conservation between related species
• Resolved a cell cluster expressing known and new druggable targets, including PAK4

## Graphical abstract

**Figure:**
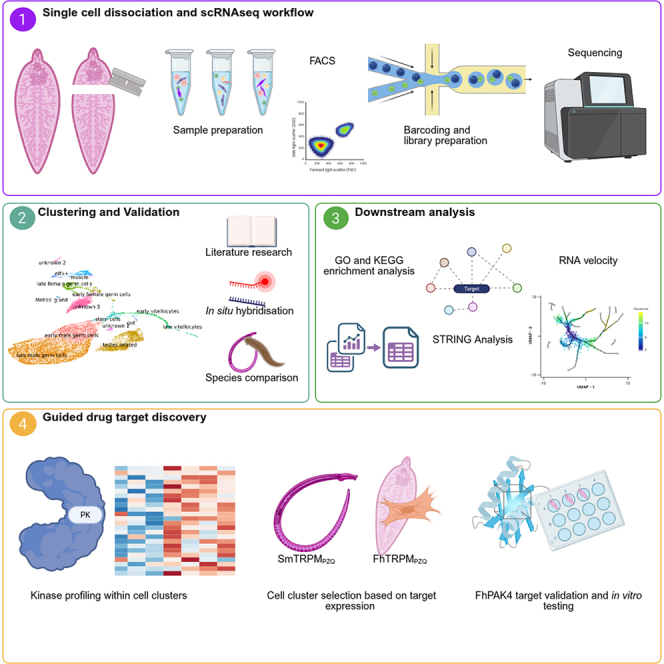

## Teaser

Molecular biology; Parasitology; Cell biology; Transcriptomics

## Introduction

Infections with parasitic helminths pose a global health challenge. As many of these diseases affect humans and animals alike, they are of major importance considering “one health” initiatives.^1^ Fascioliasis is caused by liver flukes such as *Fasciola hepatica*, a parasitic flatworm heavily affecting livestock industry. Here, it causes a huge economic burden by reducing growth and milk yield.^2^ Along with other food-borne diseases, fascioliasis is recognized by the World Health Organization as a neglected tropical disease (NTD). It is estimated that up to 17 million people are infected worldwide^3^ and 90 million are at risk of infection.^4^ The increasing number of reports on parasites being resistant to the commonly used drug triclabendazole^5^^,^^6^ and a lack of treatment alternatives or effective vaccines^7^ motivates basic research on liver flukes, aiming at the identification of drug targets.

*F*. *hepatica* has a complex life cycle, which includes an intermediate snail host and a mammalian as final host. The hermaphrodite adult worms reside within the bile duct of the final host, where they shed tens of thousands of eggs per day in order to reproduce.^8^ The eggs are released into the environment during defecation, followed by hatching of miracidia that infect snails. Within the snail, the parasite multiplies by asexual reproduction. Infected snails eventually shed cercariae, which encyst on aquatic vegetation, where they can remain infectious for months. After oral ingestion of infectious metacercariae by the final mammalian host, newly excysted juveniles (NEJs) hatch from the cysts and migrate to the liver parenchyma. There, the immature worms feed and grow into adults, which ultimately reach the bile duct where they can persist for decades, hinting at an outstanding longevity of these worms.^9^

Insights into the biology of *F*. *hepatica* have been significantly advanced by the implementation of various omics technologies.^10^ By using bulk RNA-seq, it was possible to identify genes being transcribed in different life stages,^11^ to uncover the response to anthelmintics,^12^^,^^13^ investigate interactions with the immune system,^14^ and assess the role of the nervous system in development.^15^ While being an important tool to investigate transcriptional gene expression, bulk RNA-seq naturally does not allow conclusions at single-cell resolution. Bulk analysis may also complicate the detection of rare genes or gene-expression characteristics of rare cell types due to overrepresented cell types or highly expressed genes. The advent of single-cell RNA sequencing (scRNA-seq) opened a new opportunity to investigate the molecular biology of multicellular organisms.^16^ By profiling hundreds to thousands of cells in one experiment, this technology allows the identification and characterization of cell types and their characteristic genes even on a whole-organism level, if single-cell suspensions are accessible. This untargeted method promises unprecedented insights into the molecular biology, especially of non-model organisms such as parasites, for which many other methodologies are still unavailable. In parasitic and free-living relatives of *F*. *hepatica*, specifically schistosomes and planarians, scRNA-seq technologies recently boosted the identification of cell-type markers^17^^,^^18^ and the characterization of transcription factors in developmental trajectories.^19^ By focusing on cell types with vital functions for a parasite, scRNA-seq data may also facilitate the identification of new drug targets.

@Protein kinases (PKs) have gained attention as promising drug targets in parasites such as cestodes, filaria, and other trematodes.^20^ PKs regulate most of the core biological processes in eukaryotes, like signal transduction, cell cycle or motility.^21^ Research on *Fasciola* kinases has greatly progressed in recent years, including our curation of the parasite’s kinome.^22^ Additionally, the field gained insight into the essential role of selected kinases in proliferation and development.^15^^,^^23^^,^^24^ Furthermore, we provided first evidence of druggable PKs in *F*. *hepatica*.^22^^,^^25^^,^^26^ Considerable effort was put into the development of inhibitors of human PKs for use in several diseases, leading to 80 already approved drugs^27^ and several more in clinical trials.^28^ Drug repurposing has been discussed as a valuable approach for the identification of treatment options against NTDs, as there are fewer risks involved regarding efficacy and safety considerations.^20^^,^^29^ The high number of available PK inhibitors and evidence for the druggability of kinases in parasitic worms make PKs attractive targets, potentially also for treating fascioliasis. A question to be answered is which type of PKs can we consider important for pathogen survival, e.g., based on their expression in particular cell types?

In this study, we profiled the gene expression of adult *F*. *hepatica* at single-cell resolution using the 10× chromium workflow. In order to achieve this, we established a cell dissociation protocol coupled with fluorescence-activated cell sorting (FACS) to obtain a viable cell suspension. We uncovered several different cell populations, highlighted their characteristic marker genes and predicted gene expression dynamics. Finally, the identification of several PKs with enriched expression in distinct cell types allowed us to reveal one PK with importance for parasite survival. This work presents the first single-cell data for this family of parasites and will serve as a resource for future biomedical research as well as basic understanding of pathogens.

## Results and discussion

### Determination of nuclei number of *F*. *hepatica* adults shows cell density differences along anterior-posterior axis

**Figure 1:**
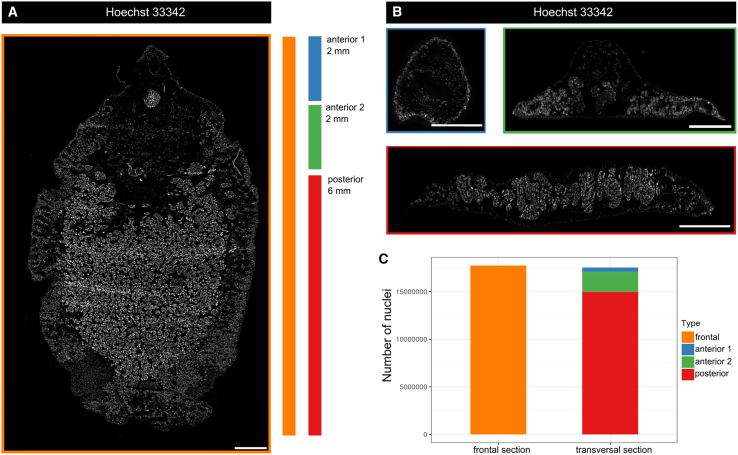
*F*. *hepatica* is structured in regions with different cellular densities (A) Representative frontal section of an adult worm with nuclei stained using Hoechst 33342. Color bars mark the worm regions selected for transversal sectioning. Scale bars: 1 mm. (B) Representative images of transversal sections with nuclei stained with Hoechst 33342. Top from left to right, sections from the anterior 1 and anterior 2 regions. Bottom: section from the posterior region. Scale bars: 1 mm. (C) Stacked bar plot indicating the extrapolated total number of nuclei derived from either frontal or transversal sections.

Basic metrics on the number of cells in multicellular organisms are helpful in planning scRNA-seq experiments, but such metrics are unknown for liver flukes. In order to determine the total cell count and to assess the distribution of cells throughout the parasite, we quantified the nuclei within sections of adult worms. To exclude a potential bias depending on the tissue area, we utilized frontal sections (Figure 1A) as well as transversal sections from representative areas of the worm (anterior part 1 and 2 and posterior part) (Figure 1B). The total number of nuclei per worm was extrapolated from the nuclei counts per section. Independent of the section plane, we arrived at a total nuclei number of around 17 million within the parasite (Figure 1C). It is to be noted that this nuclei number is only an approximation for the total cell number, as the multi-nucleated nature of the syncytial surface of the parasite, the tegument, or the fused rosette during spermatogenesis^30^ do not allow a one-to-one translation.

Compared to the two anterior parts used for quantification, most of the cells were located within the large posterior part of the worm, which contains the male reproductive tissue as well as the female vitellarium, creating a higher total nuclei count. This not only arises from the sheer size of these tissues, in addition, the male reproductive organ as well as the vitellarium also have a high cellular density (Figure 1A). This disproportional tissue and cell distribution over the body axis bears the risk that cells of the overabundant reproductive organs might overrun some of the rarer cell types, which are hence not captured in a subsequent 10× workflow. Based on the cellular composition, we therefore decided to cut the worm into two parts for subsequent scRNA-seq experiments: a posterior part and an anterior part, the latter being enriched for proportionately underrepresented cell types.

### scRNA-seq captures cells of major tissue types of *F*. *hepatica*

**Figure 2:**
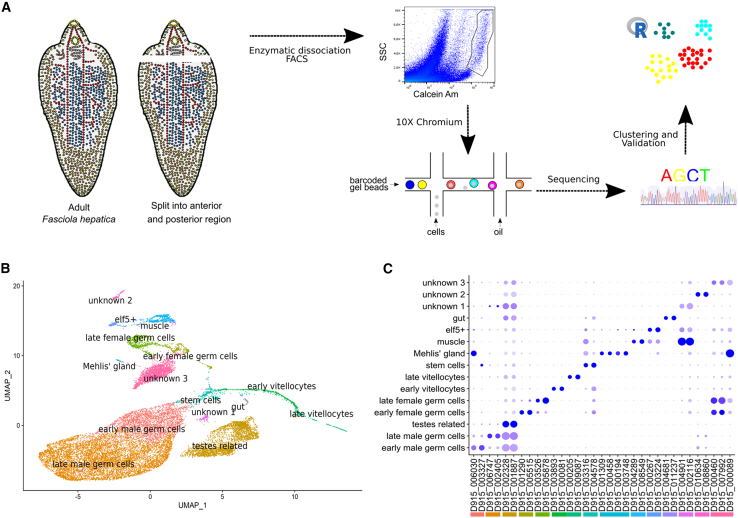
scRNA-seq allowed the classification of 15 cell clusters in adult *F*. *hepatica* (A) Schematic workflow outlining the major steps for data generation: adult liver flukes were first split into an anterior and posterior part before dissociating into single cells by mechanical and enzymatic processing. Next, cells were sorted based on the viability dye calcein. Cells were barcoded following the 10× Chromium protocol. Libraries were sequenced and clustering was carried out to identify transcriptionally distinct cells. (B) Uniform manifold approximation and projection (UMAP) of 19,581 cells. Clusters are colored and labels added. (C) Profiles of gene expression over all clusters illustrated as dotplot. Shown is the expression of at least two marker genes for each cluster, and the cluster color is indicated below each marker pair. Level of expression is indicated by color from blue (high expression) to lavender (low expression). The percentage with which the cells of a cluster express the given gene is represented by the size of the circles.

We performed scRNA-seq on cells from anterior and posterior parts of several adult individuals. First, we developed a protocol to dissociate anterior and posterior parts of the worms into single cells using a combination of mechanical and enzymatic treatment (Figure 2A). Thereafter, viable cells were enriched by FACS using calcein AM viability dye to select viable cells. By using the commercially available 10× Genomics Chromium platform, we analyzed a total of ten samples, from either anterior (4) or posterior parts (4) of worms, or whole worms (2) for comparison. Using a combination of the 10× cellranger workflow and the R package Seurat,^31^ we analyzed sequencing data of a final number of 19,581 cells. Hereby, we detected a median gene number per cell of 2,644 and on average 14,087 Unique Molecular Identifiers (UMIs) per cell after quality filtering (Table S1). Based on the total of 8,716 protein-coding genes expressed in the *F*. *hepatica* adult stage,^11^ we detected on average 30% of the total genome as transcripts per cell. This is a touch higher compared to scRNA-seq data obtained for adult *S*. *mansoni*,^18^ where a median gene number of 1,600 was detected per cell, which is 19% of the total gene count of 8,083 protein-coding genes in adult *S*. *mansoni*.^32^^,^^33^

Using Seurat, we identified 15 clusters (Figure 2B), for which distinct marker genes could be derived (Figure 2C and Table S2). In this case, a marker gene is a differentially expressed gene with the power to distinguish clusters within the presented data. The clusters were annotated with the help of published cell marker genes that are conserved across taxa, as well as by comparison to cellular markers for the closely related blood fluke *S*. *mansoni* (Table S2). We identified clusters resembling cells of the following tissues (number of clusters in brackets): muscle and muscle-associated (2), gut (1), testes (3), stem cells (1), ovary (2), vitellarium (2), Mehlis gland (1), and three clusters for which we could not find an annotation. Additional evidence for the cluster annotations of the identified clusters was gained by gene ontology (GO) and Kyoto encyclopedia of gene and genomes (KEGG) annotation and integration of the clusters into the spatial transcriptome of adult *F*. *hepatica* (Figures 4C, 4D, 5C, and S1). No clear evidence for cluster identity could be gained for the unknown clusters, as unknown 1 and 2 could not be spatially localized while unknown 3 produced diffuse signal. While cluster unknown 1 and 2 covered a limited number of markers, enrichment analysis pointed to signaling processes as a prominent feature. The various clusters, as hypothesized earlier, were not equally distributed between the anterior and posterior samples (Figure S2). Because gut, testes and vitellarium represent the largest tissues in *F*. *hepatica* and cover most of the posterior body part, the whole-worm samples and posterior samples strongly resembled each other. Specific cell types are missing in the dataset, like parenchymal or neuronal cells, which could be explained by several factors. First, rare cell types like neurons might, despite our enrichment strategy, still be overrun by overabundant cell types and not be captured during scRNA-seq. Another reason may be linked to the chosen tissue dissociation protocol, which made use of enzymatic digestion followed by flow sorting, an established procedure for other flatworms.^17^^,^^18^ One cannot fully exclude damage to more sensitive cells and/or a loss of syncytial cells, such as those of the tegument. Single-nuclei sequencing may be an elegant solution to this problem, but has so far never been applied to flatworms.

### Stem cells are characterized by expression of RNA-binding proteins and histones

**Figure 3:**
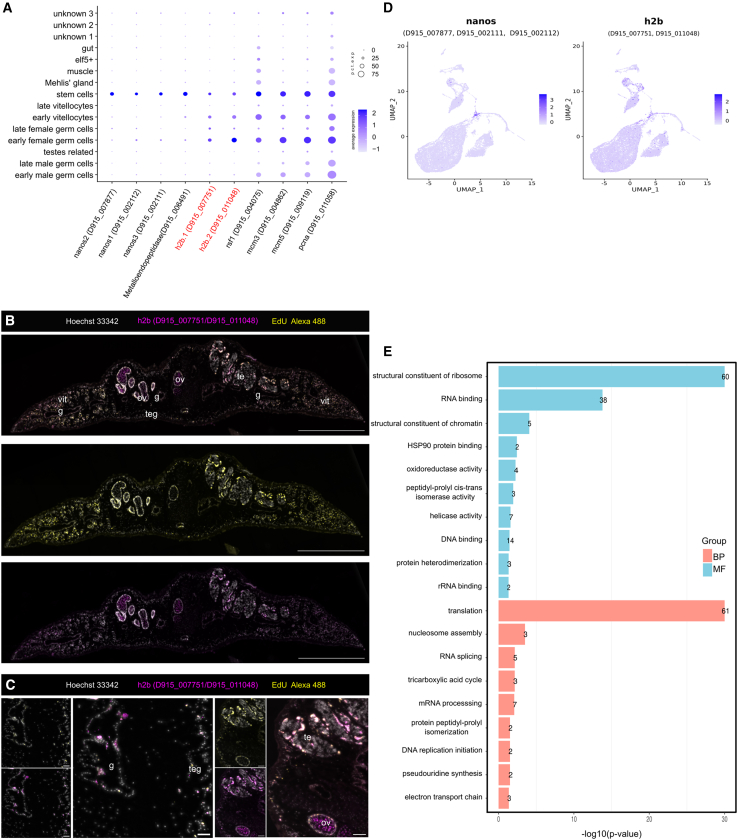
Stem cells are characterized by expression of RNA-binding proteins and histones (A) Dotplot showing the expression of stem-cell marker genes. ISH-validated genes are marked in red. Level of expression is indicated by color from blue (high expression) to lavender (low expression). The percentage with which cells of a cluster express the given gene is represented by the size of the circles. (B) Transversal section stained for *h2b* transcripts by FISH (magenta) and proliferating cells with EdU (yellow). Scale bars: 1 mm. (C) Close-up of testis and ovary (right) or proliferating cells from tegument and gut (left) from (B). Scale bars: 50 μm. (D) UMAP plot showing the merged expression of three *nanos* and two *h2b* isoforms, respectively. (E) Gene ontology analysis of marker genes (top 75% per cluster) revealed characteristic biological processes (BPs) and molecular functions (MFs). The number of enriched genes is noted at the end of each bar. Legend: gastrodermis (g), tegument (teg), testes (te), ovary (ov), vitellarium (vit).

Being a multicellular organism, the growth and development of *F*. *hepatica* depends on stem cells that give rise to progenitor cells of reproductive and somatic tissues.^34^ The remarkable output of thousands of eggs per day^8^ necessitates a massive proliferative activity of germline stem cells and differentiation of gametes. Understanding what drives that remarkable fecundity of the parasite involves understanding the gene expression controlling germline stem cells. First, the stem-cell cluster was identified based on the expression of known marker genes (Figure 3A). Among others, the cluster expressed three nanos isoforms (D915_007877, D915_002111, D915_002112) that were previously described by Robb et al. 2022^15^ for *F*. *hepatica* and are known to be stem-cell markers in *S*. *mansoni*.^35^ Another marker gene specifically expressed in the stem-cell cluster was a gene encoding a metalloprotease (D915_006491) (Figures 3A and S3). The protease was classified as an astacin-like protease based on the characteristic zinc binding motif^36^ (Figure S3). This family of proteins has been linked to activation of growth factors and processing of extracellular matrix proteins.^37^ Given recent observations on stem cells in juvenile worms,^38^ we propose a potential role for this protease in stem-cell adhesion and migration. Additionally, GO term analysis identified RNA binding and RNA processing as enriched functional terms within this cluster (Figure 3E and Table S3). RNA-binding proteins are involved in important post-transcriptional processes regulating gene expression, including mRNA processing, mRNA modification or translation.^39^ This adds an additional layer of regulation to the gene expression outcome, and functional characterization of such proteins will improve the understanding of how stem-cell proliferation in trematodes is controlled.

KEGG enrichment analysis also identified cell cycle as an enriched pathway within the stem-cell cluster (Table S4). This included genes encoding for the minichromosome maintenance (MCM) complex proteins, which are involved in DNA replication. Another marker gene for this cluster was histone 2b *(h2b*) (D915_007751), which has been directly linked to proliferation in *F*. *hepatica* juveniles.^23^ It is most likely that this cluster contains a combination of stem cells and proliferating cells from both germline and somatic origin. With the current lack of validated marker genes discriminating both cell types in liver flukes, a clear distinction in our dataset is difficult. On closer inspection, we found that marker genes of the stem-cell cluster showed two different types of expression patterns. While *nanos* expression was tightly restricted to the stem-cell cluster, other gene transcripts (e.g., *h2b*) were also detected in potential progenitor clusters of the vitelline, oogenesis and spermatogenesis lineages (Figures 3A and 3D). Transcription of *h2b* was previously used to label actively proliferating cells in schistosomes and planarians.^35^^,^^40^ To test if presence of *h2b* expression is a suitable marker for active cell proliferation in *Fasciola*, we labeled proliferating cells with the thymidine analog 5-ethynyl-2′-deoxyuridine (EdU) and stained *h2b* transcripts with fluorescence *in situ* hybridization (FISH). In *F*. *hepatica*, two more genes are annotated as *h2b* in addition to the previously mentioned D915_007751, namely D915_011048 and D915_009959. The paralogs D915_007751 and D915_011048 have 93.5% sequence similarity on nucleic acid level and show a preferential expression in stem cells and the female germline, while D915_009959 displays 79% and 78% similarity to D915_011048 and D915_007751, respectively, and is widely expressed in several different cell types (Figure 3A and Table S2). In the following, *h2b* expression refers to the expression of the two stem-cell associated genes D915_007751 and D915_011048. Because of their high sequence similarity, it is not possible to distinguish their transcriptional expression with sequence-specific probes in RNA *in situ* hybridization, which is why a combined analysis is provided (Figures 3B and 3C). We found an overlap between *h2b* positive cells and EdU positive cells in tissue sections, with around 70% of EdU positive cells (1,434 cells analyzed) being also positive for *h2b* transcripts (Figure 3C). The *h2b/*EdU double-positive cells were located in the periphery of the testicular lobes and ovary. When focusing on gonads in the tissue sections, the percentage of double-positive cells was even higher with 88% and 91% for the testes (217 cells) and ovary (311 cells), respectively (Figure 3C), which validates prior description of stem cells in that location based on histological analysis.^30^ Furthermore, *h2b* positive cells were located close to the tegument and gut tissue (Figure 3C), confirming the presence of somatic stem cells in adult flukes, as described in previous studies for juvenile worms.^34^ We also detected strong staining for *h2b* transcripts in the center of the ovary, containing mature oocytes. This can be explained by the fact that unlike stem cells, in which histone transcription is coupled to the cell cycle, both processes are decoupled during oogenesis in preparation of embryogenesis.^41^ Taken together, *h2b* transcriptional expression can serve as marker for cell proliferation in adult *F*. *hepatica*, with mature oocytes as exception.

### Gene signatures and spatial localization of different cell states during germ-cell development

**Figure 4:**
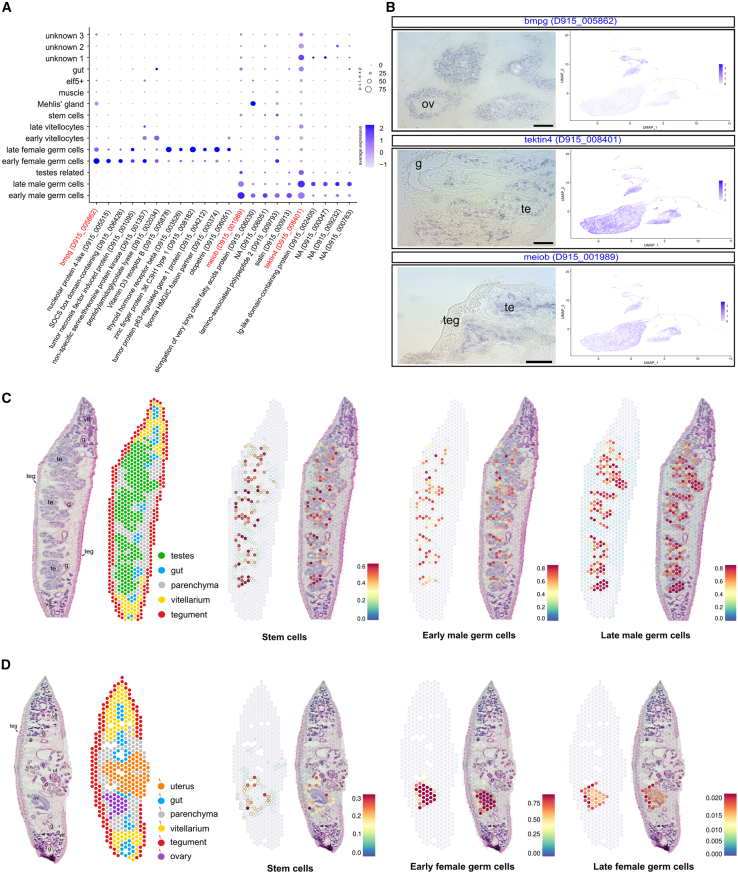
Germline cell clusters and spatial localization of different cell states during germ-cell development (A) Dotplot showing the expression of germline marker genes. ISH-validated genes are marked in red. Level of expression is indicated by color from blue (high expression) to lavender (low expression). The percentage with which cells of a cluster express the given gene is represented by the size of the circles. (B) ISH stainings (top row) for transcripts of selected male and female germline markers *bmpg*, *tektin4* and *meiob* (in blue), and corresponding UMAP plots of gene expression. Scale bars: 100 μm. (C and D) Integration of stem cell and male (C) or female (D) germ cell clusters into the spatial transcriptome.^26^ H&E stained section and spatial projection of spots covering the tissue. The color of the spots corresponds to the associated prediction scores for the clusters. Legend: gastrodermis (g), tegument (teg), testes (te), ovary (ov), uterus (ut), vitellarium (vit).

*F*. *hepatica* is a hermaphroditic flatworm, harboring both female and male reproductive tissues. We captured clusters of both tissues, which included cells related to more undifferentiated as well as more mature cells with distinct marker profiles (Figure 4A). The testes are represented by three clusters (Figure 4A). Two of the three clusters were successfully annotated and termed early and late male germ cells. Cells in the early male germ cell cluster expressed *boule* (D915_007531) and the transcription factor *one cut 1* (D915_002483), which are described to promote male germ-cell differentiation.^19^^,^^42^ Additional marker genes also hinted at cell proliferation and the initiation of differentiation as main processes in this cluster, like genes coding for histones or RNA helicases (Table S2). Furthermore, we found a strong expression of a gene annotated as meiosis specific with OB-fold (*meiob*), which is known to play a role in meiotic recombination in humans,^43^^,^^44^ within the early male germ cell cluster. Expression of *meiob* within the testes was confirmed by ISH (Figure 4B). In contrast to this, the late male germ cell cluster was enriched for genes encoding structural components like several tubulins and tektins known to be part of the sperm flagellum (Figure S4). One of the tektin genes was also shown to be strongly expressed in the testes of the worm by ISH (Figure 4B).

The ovary of *F*. *hepatica*, as the testes, is a branched organ consisting of three major cell types: the proliferating female germline stem cells and two stages of oocytes, which were further classified in early prophase oocytes and late prophase oocytes by ultrastructural analysis.^45^ While meiosis I takes place in the ovary, the oocytes undergo meiosis II only later after egg formation. In our dataset, ovarian cells were characterized by their expression of the bone marrow proteoglycan (*bmpg*), a gene characteristic for oocytes found in schistosomes.^18^ Transcripts of the *F*. *hepatica bmpg* were specifically detected in the ovary in ISH experiments (Figure 4B). We identified two clusters of oocytes, termed early and late female germ cells, based on their distinct marker gene sets, such as a vitamin D3 receptor in late female germ cells (Figure 4A).

In order to gain additional knowledge on the spatial distribution of the different germ cell clusters within the male and female reproductive tissues, we integrated the single-cell data into our recently published spatial transcriptome dataset of tissue sections from adult worms.^26^ By using the anchor-based integration method implemented in Seurat,^46^ we were able to transfer the cell type annotations into the spatial dataset, which comprises gene expression spots of 55 μm diameter obtained by 10× Visium technology. Based on this, we observed that cells of the early male germ cell cluster localized in the periphery of the testes together with cells of the stem cell cluster. Only later on, when male germ cells matured (late male germ cell cluster), they localized to the center of the testes lobes (Figure 4C). This spatial organization of cells was described earlier in histological stainings^47^ and can now be addressed on transcriptome level. In the ovary, our transcriptome data integration revealed an outer ring of stem cells again supporting the histological findings^47^ as well as our imaging data for proliferating cells (Figure 3C), while cells of the early and late female germ cell clusters were found throughout the organ (Figure 4C). This highlights the potential of this combined approach of spatial and scRNA-seq data integration to illustrate spatial relationship of cells in *F*. *hepatica*, and analyze their developmental relationships on a molecular level.

Further support for the functions associated with each cluster was obtained by GO term analysis (Table S3). The early male germ cell cluster was enriched for GO terms like RNA binding or nucleotide binding as well as several metabolic processes, further underlining a cell state undergoing cell division and growth as part of meiotic proliferation. In contrast, the late male germ cell cluster was enriched for terms involved in cytoskeletal organization, cilium assembly, and axoneme assembly (Table S3), reflecting a differentiated cell state.^30^ As for the early female germ cell cluster, GO terms related to metabolic processes, DNA binding or DNA replication were enriched in the early female germ cells cluster. In contrast, the terms enriched within the late female germ cell cluster were related to processes like signaling, phosphorylation, organization, and nuclear receptor activity (Table S3), emphasizing a complex control of signaling processes in these cells. One thyroid hormone receptor showed a highly enriched expression in the late female germ cell cluster, which suggests an important role of thyroid hormones, potentially host-derived, for germ cell maturation in *F*. *hepatica*. While it has been shown that nuclear hormone receptors are important for correct oocyte differentiation in *S*. *mansoni*,^48^^,^^49^ this class of nuclear hormone receptors has not been characterized in *F*. *hepatica* and represents an attractive topic for future studies related to host-parasite interaction.

### Identification of differentiation markers and lineage prediction of vitellocytes via RNA velocity

**Figure 5:**
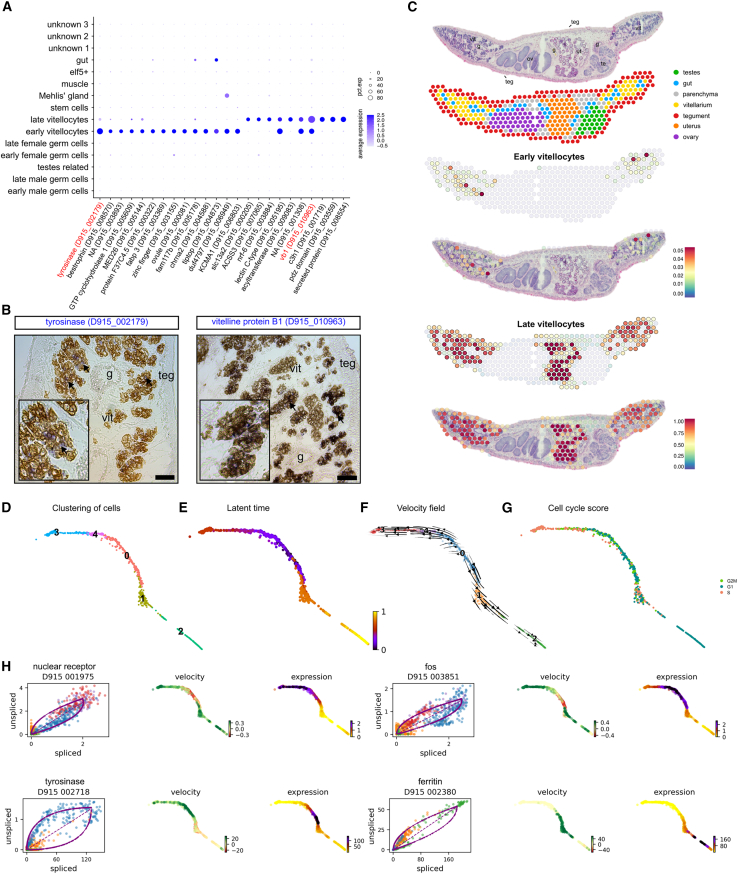
Gene expression dynamics of the vitelline lineage (A) Dotplot showing the expression of vitelline cell marker genes. ISH-validated genes are marked in red. Level of expression is indicated by color from blue (high expression) to lavender (low expression). The percentage with which cells of a cluster express the given gene is represented by the size of the circles. (B) ISH staining for transcripts of the vitelline cell markers *tyrosinase* (early vitellocytes) and *vitelline protein B1* (late vitellocytes), arrows indicate positive staining (blue). Scale bars: 100 μm. (C) Integration of early and late vitellocyte clusters into the spatial transcriptome.^26^ H&E stained section and spatial projection of spots covering the tissue is presented. The color of the spots corresponds to the associated prediction scores for the clusters. (D) UMAP of subclusters from the early and late vitellocyte clusters from the Seurat analysis. (E) Temporal relationship between cells as estimated by latent time. (F) Generalized direction of RNA velocity shown as a flow field. (G) Cell cycle scoring derived from the Seurat analysis. (H) Detailed plots for selected genes. Phase plot (left) shows proportion of spliced and unspliced reads per cell. The dashed line depicts the estimated steady state of transcription. RNA velocity plot (middle) shows the deviation of transcription from the steady state for the selected gene. Gene expression plot (right) with high expression in purple.

The vitellarium is a unique organ of flatworms, essential for the production of vitellocytes that enter ectolecithal eggs together with one oocyte. Vitellocytes (vitelline cells) are important for egg shell formation and contain nutrients for the later development of the embryo. In *Fasciola*, like in schistosomes, the immature vitelline cells heavily proliferate and develop in four different stages, called stem cells (S), intermediate types 1 and 2 (It1, It2) and mature cells (M).^50^^,^^51^ While vitellocytes and their gene expression have seen interest in schistosome research,^18^^,^^52^^,^^53^^,^^54^ no such data were available for *F*. *hepatica*, and the different cell states have been mainly categorized by their morphology.^50^ We discriminated two main clusters based on their differential gene expression, which we named early and late vitellocytes (Figure 5A). These clusters could also be localized to vitelline tissue in our spatial transcriptome.^26^ The early vitellocyte cluster was localized in the vitelline tissue, while late vitellocytes mapped to the vitelline tissue as well as the uterus, where mature vitellocytes are present within the eggs (Figure 5C). Identification of orthologs for typical genes characterizing S1 to S4 vitelline cell stages in *S*. *mansoni*^54^ suggested that the data cover the full vitelline cell lineage in *F*. *hepatica* (Figure S5). The ortholog for the nuclear receptor vitellogenic factor 1 (D915_001975) was expressed in the early vitellocytes cluster, partially coexpressing the proliferative marker *h2b*, and should represent proliferating S vitelline cells. Expression of tyrosinases (D915_002718, D915_002179) characterized an intermediate state (transition from S to It1/It2) between the early and late vitellocyte clusters, which also agrees with earlier observations in schistosomes, where maturing cells express tyrosinases but expression is absent in mature S4 cells. The expression of tyrosinase D915_002179 was additionally validated in the vitelline cells by ISH (Figure 5B). Finally, the expression of typical egg-shell genes, such as *vitelline protein B1* (D915_010963),^55^ was found in cells of the late vitellocyte cluster and as well confirmed by ISH (Figure 5B). Additionally, a ferritin (D915_002380) was highly expressed in mature vitellocytes (Figure S5), which is known to be important for egg formation in *F*. *hepatica* and other trematodes.^56^^,^^57^^,^^58^^,^^59^ Together, the investigation on marker expression suggests that the early vitellocyte cluster represents a fused population of S and It1/It2 cells, while the late vitellocyte cluster mostly represents M cells.

To verify the proposed vitelline lineage, the expression dynamics of key genes along the presumed differentiation axis from the stem cell cluster to the late vitelline cell cluster was calculated by employing RNA velocity^60^ after subclustering of cells into five clusters. RNA velocity utilizes the ratio of spliced and unspliced reads within cells over the potential lineage to predict their future cell state. Based on the inferred latent time, a clear differentiation branch with velocities directed from subcluster 0 to 1 and 2 was obtained (Figures 5D and 5E). A second branch emerged from subcluster 0 to 4 and 3 (Figure 5F), suggesting that cells in subcluster 0 undergo cell fate specification. Indeed, S vitellocytes are capable of self-renewal and may follow two different fates, either differentiation or maintaining proliferative capacity as S cells. In order to test this hypothesis, the cells were scored based on their cell cycle state (Figure 5G): the cell clusters 3 and 4 had a higher proportion of S-phase cells than the other clusters, which showed increasing proportions of cells scoring in G1 and G2/M phase (Figure 5G). The transition from S-phase dominance to G1 within the early vitellocyte cluster matches the assumption that cell fate decisions are restricted to the G1-phase, in which cells respond rapidly to differentiation signals and activate expression of developmental genes required for progression.^61^

Taken together, these observations suggest that we captured the complete vitellocyte lineage, with the self-renewing S vitelline cells representing the proliferative lineage branch (subclusters 3/4), while the differentiation lineage branch moves to It1/It2 and M cells (subclusters 1/2). This was further validated by examining the splicing dynamics of the already described marker genes. Expression of the ortholog to the schistosomal S1 cell marker vitellogenic factor 1 (D915_001975) was repressed, while tyrosinase D915_002718 was induced along the differentiation branch. Additionally, ferritin (D915_002380), showed a marked induction of expression toward S4 cells (Figure 5H). Some other characteristic marker genes like egg-shell proteins and histones could not be profiled, as they do not harbor intronic sequences. Besides the expression of already known marker genes, we discovered other genes with interesting splicing profiles (Figure S6 and Table S9). This includes the expression of a fibroblast growth receptor (D915_007307) or a fos transcription factor (D915_003851) (Figure 5H). The latter showed a marked increase in expression toward the beginning of differentiating S cells. Both of the transcription factors showed their highest expression in cluster 0 with a decreased velocity toward cluster 1.

Table S9. Top 100 dynamical genes of each cluster from RNA velocity analysis

Finally, as expected, GO terms enriched in the early vitellocyte cell cluster were related to proliferation and transcriptional activity, like nucleotide binding or RNA processing, while the late cells covered terms like iron or vitamin binding (Table S3). Overall, the existence of shared molecular vitelline-cell markers for blood and liver flukes suggests that the biological mechanisms guiding vitelline-cell function and maturation are conserved. This degree of conservation is anything but self-evident, given the highly differential sexual biology of both pathogens with liver flukes being hermaphrodites and schistosomes diecious. Insights in the reproductive biology of flatworms may allow the development of strategies to limit transmission of the parasites; consequently, the vitellarium has been discussed as a valuable target.^62^

### Genes involved in lipid metabolism are expressed in gastrodermal cells of liver flukes

As for most trematodes, the intestine of liver flukes is bifurcated with numerous branches stretching throughout the parasite’s body. The gastrodermal cells are known to express and secrete a high number of digestive enzymes, primarily cathepsins.^63^^,^^64^^,^^65^ Accordingly, we classified cells expressing known intestinal cathepsins of *F*. *hepatica*^11^ (Table S5) as gastrodermal cells (Figure S7A). These cathepsins are well established markers for the gastrodermis (Figure S7D). As to be expected, a high number of characteristic genes for this cluster associated with GO terms like proteolysis, cysteine-type peptidase activity or lipid binding (Figure S7B). Contained within the marker genes for this cluster were two genes annotated as tegumental antigen (D915_001367) and tegument-associated antigen (D915_001370). That both genes also showed non-tegumental expression in the present and our spatial data^26^ highlights the need for careful evaluation of computationally inferred gene ontology annotations, especially in non-model organisms. Genes related to lipid metabolism caught our attention, including genes coding for a phospholipase B-like protein (D915_003832), ceramide-processing enzymes (D915_008080, D915_005138), and a putative MD-2-related lipid-recognition domain-containing protein (D915_009347), the latter involved in cholesterol transport in humans.^66^ The expression of ceramide-processing enzymes matches with the presence of glycosphingolipids with a distinct ceramide composition specifically in the intestine of adult worms, which we found previously by MALDI mass spectrometry imaging.^67^ For the gene encoding phospholipase B-like protein, we confirmed specific transcript staining in the branches of the gut (Figure S7C). It can be speculated that these gut-specifically expressed genes control the lipid metabolism in gastrodermal cells of the adult worm. As liver flukes have a highly reduced lipid metabolism,^68^ processing of endogenous or host-derived lipids in gastrodermal cells warrants future investigation to improve our understanding on host-parasite interaction. At the same time, involved proteins could be interesting as anthelminthic target.

### Signaling proteins and protease inhibitors revealed in muscle cells

**Figure 6:**
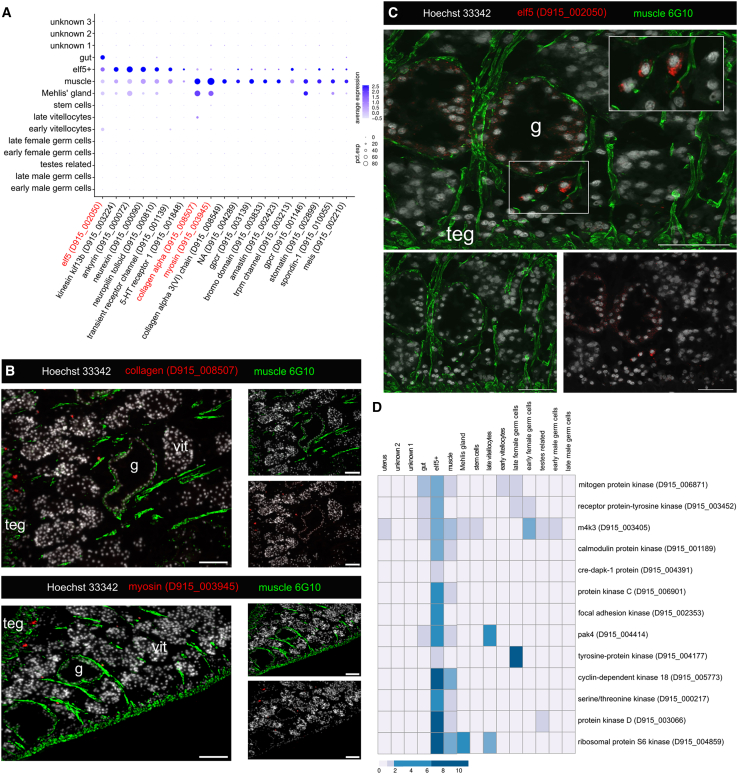
Muscle-associated cells express several PK genes (A) Dotplot showing the expression of marker genes for the muscle and elf5+ cell clusters. ISH-validated genes are marked in red (see also Figure S10). Level of expression is indicated by color from blue (high expression) to lavender (low expression). The percentage with which cells of a cluster express the given gene is represented by the size of the circles. (B) Detailed view of FISH for transcripts of *collagen* (top) and *myosin* (bottom) combined with immunolocalization of muscle fiber proteins. Scale bars: 100 μm. (C) FISH for *elf5* transcripts combined with immunolocalization of muscle fiber proteins. Scale bars: 100 μm. (D) Average expression of PK marker genes per cluster displayed as a heatmap. Note the high and enriched expression of several PKs in the elf5+ cluster. Expression values were centered and scaled for each row (each gene) individually. Legend: gastrodermis (g), tegument (teg), vitellarium (vit).

The musculature in trematodes is important for several functions essential for the fluke’s survival. The muscular system is composed of body musculature controlling the worm’s movement, the sucker musculature for attachment, as well as the muscles lining the reproductive and digestive organs.^69^ All muscles are of the invertebrate smooth type with the cell body connected to the muscle fiber via cytoplasmic connections.^62^^,^^70^ The liver fluke musculature was represented by one cluster in our data. When considering related species, a greater diversity of muscle cells would be expected,^17^^,^^18^ which could not be resolved in our data. The muscle cluster was identified by the expression of myosin and collagen as marker genes (Figure 6A) as well as conserved muscle markers from other species.^71^ The expression of collagen in muscle cells is in agreement with studies in planarians and schistosomes,^18^^,^^72^ which described muscle cells as a main source of extracellular matrix. We further validated the expression of collagen and myosin by ISH. As a reference, we combined FISH with immunofluorescence, utilizing an antibody against muscle fiber protein frequently used in planarian research.^73^ Cells expressing collagen and myosin were widely distributed and located in close proximity to the stained muscle fibers in both the subtegumental muscle layer and throughout the body (Figures 6B and S8). This pattern clearly fits the nature of flatworm muscle fibers described above. Also among marker genes, we found central regulators of cell signaling expressed in a proportion of muscle cluster cells. Among these were PK C, G-protein coupled receptors and phosholipase C (Figure 6A and Table S2). For the latter, signaling in response to FMRFamides was previously suggested for *Fasciola* muscle fibers.^74^ A small fraction of muscle cells also expressed the 5-HT receptors 1 (D915_001848) (Figure 6A), a class of receptor which is thought to be involved in the serotonin-dependent activation of muscle fibers in flatworms.^75^^,^^76^ Unexpectedly, expression of the cystatin like stefin-2 (D915_009861) was high within the muscle cluster. Stefins act as inhibitors of cysteine proteases, such as cathepsins, and previous work on *F*. *hepatica* showed the localization of stefins within gastrodermal cells, the tegumental area as well as the reproductive organs.^77^ Our data suggest that the transcripts for stefin-1 (D915_009335) were indeed present in cells of the reproductive organs as well as in the gut, while stefin-2 (D915_009861) and stefin-3 (D915_001085) were also present in the muscle cluster, a cell type for which expression of these protease inhibitors has not been described before (Figure S9). We speculate that these stefins counterregulate the activity of muscle-associated cysteine proteinases, like D915_010977, which is among the marker genes (Table S2).

### Discovery of elf5+ cells associated with muscle cells, protein kinase signaling, and cell adhesion

An additional cluster shared some markers with the muscle cluster, but featured several distinct marker genes (Figure 6A). We termed this cluster elf5+ based on the expression of the ets transcription factor elf5 (D915_002050), which was shown to regulate extracellular matrix composition in planarians.^78^ In order to locate these elf5+ cells within *F*. *hepatica*, we performed FISH. Cells positive for *elf5* transcripts located in close contact with muscle fibers and were found underneath the tegument (Figure S10A) but also scattered within the fluke’s body (Figure 6C). This was confirmed further by the spatial integration of this cluster, which mapped the elf5 cluster to some of the tegumental spots (Figure S1). While the muscle cluster appeared to be more involved in metabolic processes, translation and transport, the elf5+ cluster was enriched in GO terms related to cell communication and signal transduction (Table S3). This was further supported by KEGG analysis showing enrichment of pathways like extracellular matrix-receptor interaction, Ras signaling as well as focal adhesion or MAPK signaling (Table S4). Related to these terms are for example the neuropilin and tolloid protein (D915_000810), which has been found in neurons as well as a type of smooth muscle cells in humans^79^ and functions in the neuromuscular junction in *Drosophila melanogaster*,^80^ or two genes encoding for ankyrin 2 (ankyrin B) (D915_000071, D915_002954). Ankyrin 2 was shown to bind to spectrin beta, which was also among the marker genes of elf5+ cells and muscle cells (D915_003953).^81^ The spectrin-ankyrin interaction is involved in the attachment and linking of membrane channels, receptors and transporters to the cytoskeleton, and especially type 2 ankyrins have been shown to function in nerve and muscle cells.^82^^,^^83^ In addition, three genes encoding ankyrin repeat domain-containing proteins were among the elf5+ cluster marker genes. The human ankyrin repeat domain 2 (ANKRD2) protein plays a role in the stretch-response of muscle cells. Despite this association of several genes in the elf5+ cluster with muscle cell function and the close proximity of elf5+ transcripts to muscle fibers within tissue sections, elf5+ cells seem to be of non-muscle nature. In double FISH experiments, all elf5+ cells were negative for collagen transcripts (Figure S10A). Additionally, staining for elf5 transcripts combined with synapsin immunolocalization showed an association with nerve structures, but we could not clearly identify elf5 positive cells as nerve cells (Figure S10B).

Further GO terms were linked to the transport and binding of calcium, such as calmodulin-regulated spectrin-associated protein 1-B (D915_005852) (Table S3). In this context, an interesting feature of a proportion of the elf5+ cells is the high expression of the transient receptor potential cation channel TRPM_PZQ_ channel (D915_003213) (Figure 6A), a calcium channel revealed as pan-trematode drug target but with still unknown role in parasitic flatworms. Recent data by the Marchant lab suggested that this channel is activated in a ligand-independent manner by membrane stretch.^84^ Ligands targeting the schistosomal TRPM_PZQ_ (praziquantel) or the *Fasciola* TRPM_PZQ_ (BZQ) caused tegumental damage,^85^^,^^86^ which fits to the localization of elf5+ cells in close proximity underneath the tegument.

Further STRING analysis of elf5+ markers provided additional evidence for the enrichment of functional networks including components of focal adhesion complex (Figure S11), which acts as adapter at the interface between the plasma membrane and the actin cytoskeleton and mediates mechano-transduction. Paxillin interacts with talin and guanine nucleotide exchange factors, while talin also binds tensin-3 and Rho GAP proteins.^87^ Genes encoding for all five proteins were found as markers of the elf5+ cluster (D915_006875, D915_001787, D915_001504, D915_000437, D915_000158), which altogether argues for a type of focal adhesion signaling characteristic for elf5+ cells. Additionally, functional network enrichments revealed by STRING were related to activity of glutamate kainate receptors, or signaling by receptor tyrosine kinases. All in all, co-expression of the stretch-sensitive TRPM_PZQ_ channel, various ankyrin-repeat proteins related to the membrane skeleton, and core proteins of focal adhesions may point to a mechano-sensing function of elf5+ cells.

To characterize this cell type in more detail, we focused on cell signaling-associated genes and the expression of a notable number of PKs predicted by STRING, which are central regulators of a plethora of cellular process.^21^ A total of 16 PKs was found among the marker genes (Figure 6D and Table S2). These included two proposed PK C (PKC) genes, D915_006901 and D915_003066; although annotated as PKC, D915_003066 shows closer sequence similarity to PK D and was termed accordingly. PKC was expressed in the tegumental/subtegumental area in our spatial transcriptome data,^26^ matching the localization of elf5+ cells described above. Other highly characteristic kinases were the focal adhesion kinase (D915_002353) and the p21-activated kinase 4 (PAK4, D915_004414). These kinases are typically involved in signaling related to extracellular matrix binding and focal adhesions.^88^^,^^89^

### An inhibitor of the p21-activated PAK4 kinase reduces fluke vitality

PKs are well druggable targets, also in various helminths.^90^ The important role of PKs in the formation and progression of cancer has led to the development of small-molecule inhibitors, which have potential for drug repurposing against parasites.^20^ The enrichment for various PKs within the elf5+ cluster as well as the expression of the pan-flatworm target TRPM_PZQ_ led us to the hypothesis that the cells within the elf5+ cluster might be valuable for the identification of drug targets with vital functions for the parasite.

**Figure 7:**
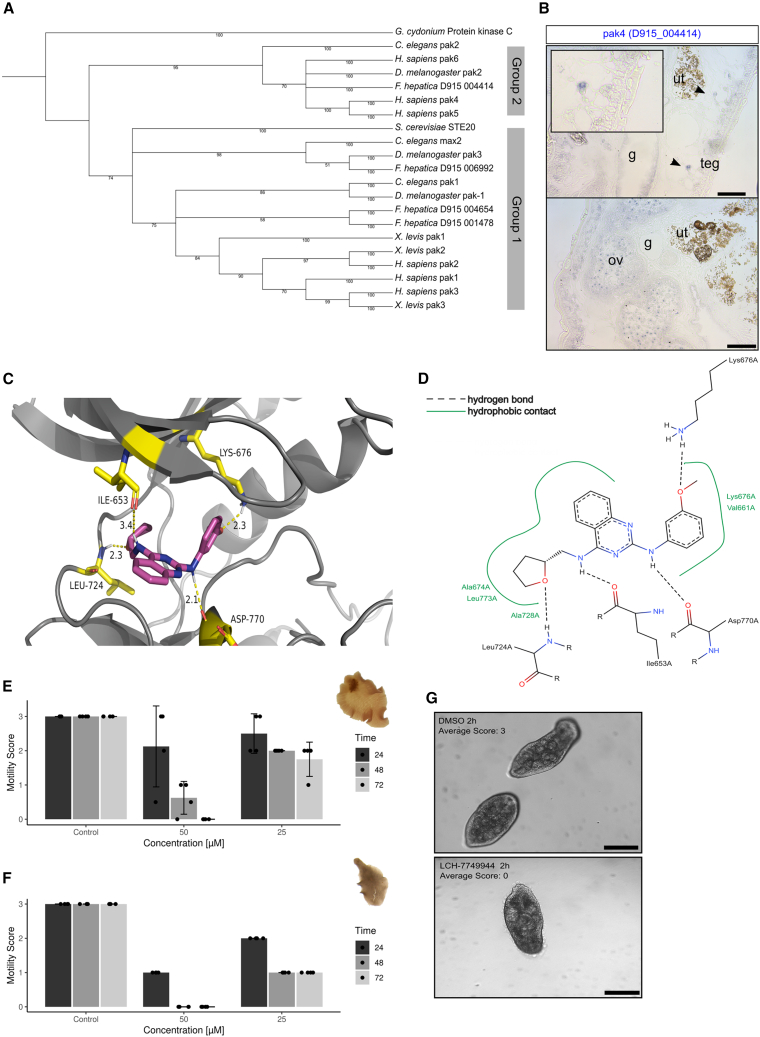
An inhibitor of the p21-activated PAK4 kinase reduces parasite vitality (A) Phylogenetic tree of PAK orthologs of *F*. *hepatica* and other species (accession numbers see Table S6). p21 families are indicated, PKC from *Geodia cydonium* served as outgroup. (B) ISH staining for Fh*pak4* transcripts in the subtegumental region and within female germ cells. Scale bars: 100 μm. (C) Representation of LCH-774994 (stick representation with carbons in magenta) in FhPAK4 (gray), interactions with Ile653, Lys676, Leu724, and Asp770 (shown in yellow sticks) are depicted in yellow dashed lines with their interaction distance in angstroms (Å). (D) 2D representation of LCH774994 interacting with the residues in the binding pocket of FhPAK4. (E and F) Motility scores of adult worms (E) and immature worms (F) under varying LCH-7749944 concentrations over 72 h (four replicates per condition). Error bar shows standard deviation. (G) NEJs after 2 h treatment with 50 μM of LCH-7749944 (bottom) compared to vehicle-treated NEJs (top). Representative images of three worms per condition are shown. Statistical differences to control were determined using Mann-Whitney U test, *p* values below 0.05 are indicated with ∗. Scale bars: 200 μm. Legend: gastrodermis (g), tegument (teg), ovary (ov), uterus (ut).

We selected PAK4 as a candidate kinase (D915_004414). PAK4 represents a still unexplored kinase in trematodes, but has shown promise in the treatment of various forms of cancer.^91^ PAK4 is a member of the p21-activated kinase (PAK) family, which is characterized by a p21-binding domain (PDB), also called Cdc42/Rac interactive binding (CRIB) motif.^92^ In humans, PAK4 is involved in a variety of cellular processes, including cell migration and cytoskeletal reorganization, and undergoes nucleo-cytoplasmic shuttling.^93^^,^^94^ In invertebrates, PAK proteins play roles in the development of the central nervous system of the fruit fly *D*. *melanogaster*,^95^ while for *Caenorhabditis elegans*, they are important for axon guidance, gonad development and mechanotransduction.^96^^,^^97^^,^^98^ Most organisms have multiple PAK family members, which belong to two groups. Group 1 and group 2 PAKs differ in their structure as well as their function.^92^^,^^99^ Within the genome of *F*. *hepatica*, we detected three more PAK kinases (Figure S13A), based on the presence of the characteristic N-terminal PDB domain. By comparing *F*. *hepatica* PAK sequences with sequences from model organisms, FhPAK4 could be identified as a group 2 family member, while the other PAKs allocated to group 1 (Figure 7A). Based on these results, we termed D915_001478 and D915_004654 as FhPAK1 and FhPAK2, respectively. The last homolog, D915_006992, clustered within a previously described group^96^^,^^100^ together with the more divergent PAK members *D*. *melanogaster* PAK3 and *C*. *elegans* MAX-2, which is why we named this kinase FhPAK3. While *fhpak1* and *fhpak2* were transcribed in several different cell types, *fhpak3* was expressed abundantly in mature oocytes and *fhpak4* expression was confined to the elf5+ cluster, the gastrodermis and vitellocytes (Figure S13B). Chromogenic ISH experiments localized *fhpak4* transcripts in oocytes, in large cells scattered underneath the tegument (Figure 7B) as seen for *elf5* transcripts, as well as weakly in the gastrodermis. The presence of *fhpak4* transcripts within the nucleus of oocytes might hint at a role for FhPAK4 in early embryo development. In line with this hypothesis is the high abundance of transcripts within the eggs of *F*. *hepatica* that we found in available bulk RNA-seq data.^11^ Maternal transcripts of *pak4* were also detected in the developing embryo of zebrafish, where PAK4 is essential for myelopoiesis.^101^ Because PAK kinases have not previously been studied in parasitic helminths, we profiled the proteomic data of a range of parasitic flatworms. Based on the conserved domain architecture of an N-terminal PDB domain as well as a kinase domain, we detected a total of four PAK kinases in all profiled worms. Next, based on pairwise global sequence alignments, we compared all parasitic as well as model organism PAKs (Figure S12 and Table S10). For group 1 PAKs, alignments revealed identities of around 70% in domain sequences and between 30% and 40% in full length sequences (Figure S12). Surprisingly, FhPAK4 was more similar to human PAK4, with an overall sequence identity of 30.8% over the full-length sequence and 64.1% in their kinase domain (Table S10), than to other parasitic kinases, which could point to an evolutionary divergence of the FhPAK4. The high sequence identity between FhPAK4 and the human counterpart made this enzyme an interesting target for a drug repurposing approach.

Table S10. Pairwise identity of PAK kinases

Due to the numerous roles of the human PAK4 in various cancer types, small molecule-inhibitors have been developed.^102^^,^^103^ We evaluated the efficacy of the commercially available ATP-competitive inhibitor LCH-7749944, which was shown to have an impact on proliferation, migration and invasion of gastric cancer cells.^104^ LCH-7749944 has seen wide use in the inhibition of PAK4 in various tissue and cell types.^105^^,^^106^^,^^107^^,^^108^ The inhibitor was shown to have a specific effect on human PAK4 and has been used in concentrations up to 30 μM in cell culture experiments.^109^ Additionally, LCH-7749944 was shown to have high affinity to PAK4 in activity assays.^104^ The binding mechanism of LCH-774994 was predicted to involve interaction with residues within the hinge/P loop regions of the human PAK4.^110^ We found high sequence similarities between human and FhPAK4 for both regions, while the three other *Fasciola* PAK proteins diverged to a higher degree (Figure S14 and Table S10). In order to further explore the possible binding mode of LCH-7749944 to the *Fasciola* PAK4 protein, we performed molecular docking of the inhibitor into the ATP binding pocket of FhPAK4 that was modeled based on the human PAK4 PDB structure (ID: 2CDZ). Our results predicted a hydrogen bond at the Leu724 position, which corresponds to a similar bond found at Leu398 in the human kinase. Other polar interactions that were predicted involved Ile653, Lys676, and Asp770 residues (Figures 7C and 7D). The carbonyl backbone of Ile653 was predicted to form a weak hydrogen bond (at 3.4 Å distance) with the NH group between the tetrahydrofuran ring and quinazoline core of the ligand at the β-sheet I region. The side chain protonated amino group (-NH3^+^) of Lys676 at the β-sheet III region is in hydrogen-bonding distance (2.3 Å) from the oxygen of the anisole moiety of the ligand. Similarly, the oxygen of the tetrahydrofuran ring is close enough to the α-amino group of Leu724 at the hinge region for a hydrogen bond (2.3 Å). The NH group between the quinazoline core and the anisole moiety of LCH-7749944 and the carbonyl backbone of Asp770 forms a hydrogen bond (2.1 Å) within the catalytic loop region of FhPAK4. Such interactions suggest reasonable complementarity of LCH-7749944 to the FhPAK4 binding site, and the predicted model is consistent with the experimental finding that LCH-774994 can function as an ATP-competitive inhibitor against human PAK4. Despite this evidence it has to be pointed out that, due to the sequence overlap of the different *Fasciola* PAK proteins, LCH-774994 might additionally target other PAKs next to FhPAK4.

To identify the potential of FhPAK4 as drug target, we tested this inhibitor against different disease-relevant parasite stages: NEJs found in the host’s intestine, immature worms migrating and feeding through liver tissue, and the bile duct-residing adult worms. For all three stages, we observed a drastic reduction of the motility during *in vitro* culture after LCH-774994 treatment. Adult worms showed moderate reduction of motility after incubation with 25 μM, but were severely affected with 50 μM after 48 h, ending in 100% lethality after 72 h (Figure 7E). For immature worms, treatment led to a strong reduction of motility with 50 μM already after 24 h, and all worms died after 48 h (Figure 7F). Thus, immature worms appeared more susceptible compared to adults. NEJs were even more sensitive, since all died within a few hours of exposure to 25 μM, displayed impaired tissue integrity and prominent lesions on their tegument (Figure 7G). Previous research highlighted the druggability and role of PAKs in human host cells, for example when it comes to host-cell invasion or pathogen-induced manipulation of the host’s cytoskeleton by viruses, bacteria and parasites.^111^ Here, we provide insight that PAK also of pathogen origin is essential for pathogen survival and appears attractive for therapeutic approaches. Thus, the example of FhPAK4 illustrates that the elf5+ cells may be of high interest with respect to drug development.

To conclude, we present the first transcriptome for *F*. *hepatica* at single-cell resolution. The scRNA-seq dataset covers several cell types that are important for proliferation and reproduction, as well as somatic cell types important for parasite vitality, like gastrodermal cells and cells of the musculature. The identification of molecular markers characteristic for each cell type delivered information on cell-type specific functions. We described a previously unrecognized muscle-associated cell type, characterized by enriched expression of TRPM_PZQ_, focal adhesion components, and a multitude of PKs. By prioritizing the family of p21-activated kinases within this cell type, we highlighted the usefulness of this scRNA-seq dataset in the discovery of druggable targets. Thus, this dataset can serve as a resource for addressing basic and applied research questions, by providing valuable insights in the cellular biology of a multicellular pathogen.

### Limitations of the study

We report the transcriptional characterization of several cell types. Due to the abundance of cells of the male reproductive tract, this dataset misses some of somatic cell types, like cells of the parenchyma, tegument, mature spermatozoa, excretory system or the nervous system. Alternative ways to enrich such cell types would add to this dataset.

## Resource availability

### Lead contact

Further information and requests for resources and reagents should be directed to and will be fulfilled by the lead contact, Simone Haeberlein (simone.haeberlein@vetmed.uni-giessen.de).

### Materials availability

Plasmids for ISH probe synthesis reported in this study are available from the lead contact upon request.

### Data and code availability

• Data: All raw sequence data were deposited in the SRA under the project accession PRJNA1073729. Accession numbers are listed in the key resources table. Filtered feature barcode matrices have been deposited at Zenodo (https://doi.org/10.5281/zenodo.17780131). DOIs are listed in the key resources table. Analyzed data can be visualized and explored in Cirrocumulus using the following link: https://www.uni-giessen.de/haeberlein-lab/en/info.
• Code: This study does not report original code.
• Additional information: Any additional information required to reanalyze the data reported in this study is available from the lead contact upon request.

## Acknowledgments

The authors acknowledge the use of de.NBI cloud and cloud computing at the Bielefeld-Giessen Resource Center for Microbial Bioinformatics (BiGi) of the University of Giessen. Financial support by the 10.13039/501100001659Deutsche Forschungsgemeinschaft (DFG) under grant HA 6963/2-1 and by the State of Hesse, LOEWE Center DRUID (LOEWE/1/10/519/03/03.001(0016)/53), is gratefully acknowledged. O.P. received a scholarship by the Justus Liebig University Giessen. The Graphical Abstract was created in BioRender. Puckelwaldt, O. (2026) https://BioRender.com/c4urhhs.

## Author contributions

Methodology, O.P.; investigation, S.G., O.P., S.A., J.K., and J.S.; visualization, O.P., S.G., and J.S.; writing – original draft preparation, O.P.; writing – review and editing, all authors; funding acquisition, C.S. and S.H; supervision, P.K. and S.H.; conceptualization, O.P. and S.H.

## Declaration of interests

The authors declare no competing interests.

## STAR★Methods

### Key resources table

**Table:** 

REAGENT or RESOURCE	SOURCE	IDENTIFIER
**Antibodies**		

Anti-Digoxigenin-POD, Fab fragments	Roche	Cat#11207733910; RRID: AB_514500
Anti-Digoxigenin-AP, Fab fragments	Roche	Cat#11093274910; RRID: AB_514497
6G10-2C7 (Muscle antibody)	Developmental studies Hybridoma Bank	RRID: AB_2619613
3C11 (Synapsin antibody)	Developmental studies Hybridoma Bank	RRID: AB_528479
goat anti-mouse IgG	Thermo Fisher	Cat#A32723; RRID: AB_2633275

**Bacterial and virus strains**		

NEB 10-beta Competent *E*. *coli* (High Efficiency)	New England Biolabs	Cat#C3019H

**Chemicals, peptides, and recombinant proteins**		

RPMI 1640 Medium	Gibco	Cat#21875034
Chicken Serum	Gibco	Cat#16110082
Antibiotic-Antimycotic	c-*c*-pro	Cat#Z-18-M
Tissue-Tek O.C.T. Compound	Sakura Finetek	Cat#4583
Q5 High-Fidelity DNA Polymerase	New England Biolabs	Cat#M0491L
AccuPrime Taq DNA-Polymerase, High Fidelity	Invitrogen	Cat#12346086
T4 DNA Ligase	New England Biolabs	Cat#M0202L
AhdI	New England Biolabs	Cat# R0584L
T3 RNA polymerases	Roche	Cat#11031163001
SP6 RNA polymerase	Roche	Cat#11487671001
Digoxigenin-11-UTP	Roche	Cat#11209256910
BCIP	Roche	Cat#11383221001
NBT	Roche	Cat#11383213001
LCH-774994	Selleckchem	Cat#S1976
Trypsin	Sigma-Aldrich	Cat#T4549
DNAase 1	Sigma-Aldrich	Cat#DN25
HBSS	Sigma-Aldrich	Cat#H6648
Calcein AM	Invitrogen	Cat#C1430

**Critical commercial assays**		

Chromium Next GEM Single Cell 3' GEM, Library & Gel Bead Kit v3.1	10× Genomics	Cat#1000147
TSA Cyanine 3 System 50–150 slides	Akoya	Cat#NEL704A001KT

**Deposited data**		

*F*. *hepatica* genome	WormBase ParaSite (WBPS17/18)	PRJNA179522
Raw sequencing data	BioProject	PRJNA1073729
3D Structure PAK4	Eswaran et al.^112^	PDB: 2CDZ
Ligand structure LCH-7749944	PubChem	ID: 2951910

**Experimental models: Organisms/strains**		

Wistar rats	Janvier Labs	RjHan:WI
*Fasciola hepatica*, Italian strain	Ridgeway Research	https://ridgewayresearch.co.uk/parasite-diagnostics-laboratory/available-parasites/

**Oligonucleotides**		

Forward and reverse primers for riboprobe synthesis	This paper	Table S6
PJC53.2 sequencing primer 5’-TTCTGCGGACTGGCTTTCTAC-3′	Li et al.^113^	N/A
T7 extended primer 5′-CCTAATACGACTCACTATAGGGAG-3′	Beutler et al.^114^	N/A

**Recombinant DNA**		

PJC53.2 (Backbone/TA Cloning Vector)	Collins et al.	Addgene#26536
Fhbmpg_PJC53.2	This paper	Plasmid#OP14; D915_005862
FhTyrosinase_PJC53.2	This paper	Plasmid#OP21; D915_002718
Fhmyosin_PJC53.2	This paper	Plasmid#OP30; D915_003945
FhVB1_PJC53.2	This paper	Plasmid#OP33; D915_010963
Fhmeiob_PJC53.2	This paper	Plasmid#OP42; D915_0001989
Fhtektin_PJC53.2	This paper	Plasmid#OP39; D915_0008401
FhCathepsinL_PJC53.2	This paper	Plasmid#OP03; CL2
Fhpak4_PJC53.2	This paper	Plasmid#OP50; D915_004414
Fhelf5_PJC53.2	This paper	Plasmid#OP83;D915_002050

**Software and algorithms**		

Seurat (v4.3.0.)	Hao et al.^115^	https://satijalab.org/seurat/
InterProScan (v5.60.92.19)	Jones et al.^116^	https://ftp.ebi.ac.uk/pub/software/unix/iprscan/5/5.60-92.0/interproscan-5.60-92.0-64-bit.tar.gz
topGO (v2.46.0)	Alexa and Rahnenfuhrer.^117^	https://bioconductor.org/packages/release/bioc/html/topGO.html
pheatmap (v1.0.12)	Kolde	https://github.com/raivokolde/pheatmap
SMART	Letunic et al.^118^	http://smart.embl-heidelberg.de/
Cell Ranger (v7.0.0)	10xGenomics	https://www.10xgenomics.com/support/software/cell-ranger
clusterProfiler (v 4.18.4)	Yu et al. 2012.^119^	https://bioconductor.org/packages/release/bioc/html/clusterProfiler.html
Fiji	Schindelin et al.^120^	https://fiji.sc/
MrBayes (v3.2.7)	Ronquist et al. 2012.^121^	https://nbisweden.github.io/MrBayes/
MAFFT (v7.505)	Katoh et al.^122^	https://mafft.cbrc.jp/alignment/software/
MUSCLE (v5.1)	Edgar.^123^	https://www.drive5.com/muscle/
Interactive tree of life	Letunic and Bork.^124^	https://itol.embl.de/
velocyto (v0.17alpha)	La Manno et al.^125^	https://velocyto.org/velocyto.py/index.html
scVelo	Bergen et al.^60^	https://scvelo.readthedocs.io/en/stable/installation.html
EMBOSS (v 6.6.0.0)	Rice et al.^126^	http://emboss.open-bio.org/
seqkit (v 2.12.0)	Shen et al. 2016.^127^	https://github.com/shenwei356/seqkit
Swiss-Model	Waterhouse et al.^128^	https://swissmodel.expasy.org/
AlphaFold2	Jumper et al.^129^	https://alphafold.ebi.ac.uk/
TLDR webserver	Irwin et al.^130^	https://tldr.docking.org/
OpenBabel (v3-1-1)	O’Boyle et al.^131^	https://github.com/openbabel/openbabel
AutoDock Vina (v1.1.2)	Eberhardt et al.^132^	https://github.com/ccsb-scripps/AutoDock-Vina
UCSF ChimeraX	Petersen et al.^133^	https://github.com/RBVI/ChimeraX
PyMol	PyMOL	https://www.pymol.org/
ProteinPlus Webserver	Schöning-Stierand et al.^134^	https://proteins.plus/

**Other**		

Selected files of this work, including the seurat object, filtered feature barcodes, code and files for the pathway analysis have been made available in Zenodo: https://doi.org/10.5281/zenodo.17780131.		

### Experimental model and study participant details

#### Animal model and ethical statement

Animal experiments using rats (*Rattus norvegicus)* as model hosts for *Fasciola hepatica* were performed in accordance with Directive 2010/63/EU on the protection of animals used for scientific purposes and the German Animal Welfare Act. The experiments were approved by the Regional Council (Regierungspraesidium) Giessen (V54-19c20 15 h 02 GI 18/10 Nr. A16/2018).

#### Biological material

Infectious metacercariae of an Italian *F*. *hepatica* strain were purchased from Ridgeway Research (UK). In order to obtain adult worms, male Wistar rats RjHan:WI (Janvier) aged 4–5 weeks were orally infected with 25 metacercariae. Rats were sacrificed by cervical dislocation after CO_2_ anesthesia at 12–14 weeks post infection (p.i.) to obtain adult flukes, or at 4 weeks p.i. for immature flukes. Worms were kept in RPMI1640 media containing 10% chicken serum (Gibco, Thermo Fisher Scientific, Germany) and 1% ABAM (10,000 units penicillin, 10 mg streptomycin, and 25 mg amphotericin B per milliliter) (c.c.pro, Germany) for 2–3 h, to allow clearance of gut contents. Adult worms were then used further for ISH processing, inhibitor treatment, or cell dissociation, while immature worms were used for inhibitor tests only. NEJs were obtained by excystment of metacercariae as described by McVeigh et al. 2014^135^ and used for inhibitor tests after a 24 h rest period at 37 °C and 5% CO_2_.

### Method details

#### Determination of total nuclei number

To determine the total nuclei number for adult worms, we used 10 μm thick cryosections with either frontal or transversal sectional plane and stained them with 1 μg/mL Hoechst 33342. Transversal sections were prepared from different regions, either from the area between oral sucker and ventral sucker (anterior 1), between ventral sucker and uterus (anterior 2), or the area posterior to the uterus (posterior). For counting of nuclei, we used a Fiji^120^ thresholding approach. We first subtracted the background using a sliding paraboloid method. Next, we set a threshold to select single nuclei, binarized the image and applied watershedding to split merged nuclei. Total nuclei were counted by the “Analyze Particles” option. For each region, four separate sections were counted and the mean number of nuclei was then multiplied by a factor derived from the thickness of the respective tissue area divided by the section thickness. For calculation of total nuclei numbers based on three frontal sections, the multiplication factor derived from the total worm thickness divided by the section thickness.

#### Single cell dissociation

Prior to dissociation, ∼20 worms were separated into an anterior and posterior part by cutting with a razor blade behind the posterior end of the uterus. Anterior and posterior parts were kept separate until stated otherwise. Afterward, the tissue was minced using a razor blade. The tissue was flushed into a 15 mL tube using 10 mL digestion buffer containing 5 × trypsin (Sigma-Aldrich, Germany), 1 mM EDTA and 10 mg/mL DNAse I (Sigma-Aldrich, Germany) in HBSS without calcium and magnesium (Gibco, Thermo Fisher Scientific, Germany). Single cells were liberated in dissociation buffer for 45 min at 37 °C on a thermoshaker. The mixture was frequently agitated using a 1 mL pipet utilizing a wide bore tip. The reaction was stopped by adding equal volume of 2% BSA in HBSS with 1 mM EDTA.^136^ The solution was passed through 100 μm and 40 μm cell strainers and subsequently washed by centrifugation at 400*g* at 4 °C for 10 min. The cells were washed two more times for 5 min using 10 mL 2% BSA in HBSS with 1 mM EDTA at 4°C. After Calcein AM (Invitrogen, USA) staining, single viable cells were sorted using a S3e Cell Sorter (Bio-Rad, USA). The whole procedure took around 3 h until cells were ready for subsequent steps.

#### Library preparation and sequencing

Prior to proceeding with library preparation, the cells were centrifuged at 400 g and 4 °C for 5 min and resuspended in 0.2% BSA in PBS. Library preparation was carried out using the Chromium Next GEM Single Cell V3.1 Kit from 10× Genomics. Cells were prepared and loaded on the Chromium controller and library preparation was carried out following the manufacturers’ guidelines. The resulting libraries were sequenced on either a Illumina NextSeq 2000 or NovaSeq 6000 system.

#### Analysis of scRNA-seq data

Sequencing data was mapped onto the *Fasciola* genome obtained from WormBase Parasite release 17 (PRJNA179522) using Cell Ranger (version 7.0.0). Prior to mapping, the mitochondrial genome of *F*. *hepatica*^137^ (available under GeneBank accession number: AF216697) was added to the genome (modified genome version available upon request). Further analysis was performed using the software package Seurat version 4.1.1 (https://satijalab.org/seurat/). The samples were filtered to only contain cells with more than 1000 UMI and less than 50,000 UMI (doublet exclusion), more than 750 Features, and a percentage of mitochondrial reads less than 5%. Summarized quality metrics are shown in Table S1. Samples were then normalized using log normalization in the NormalizeData function and 1500 variable features were identified using the FindVariableFeatures function with the “vst” method. From here, the samples belonging to separate groups (anterior, posterior or whole worm) were integrated separately using a combination of FindIntegrationAnchors and IntegrateData. Afterward, the groups were integrated using the same methodology. After scaling the data with the ScaleData function, RunPCA, and RunUMAP(reduction = “pca”, dims = 1:25, n.neighbors = 30, seed.use = 42) were used to further process the data. The number of principal components was identified using a combination of Elbowplot and JackStraw. Clustering was done by FindNeighbors(reduction = “pca”, dims = 1:25) and FindClusters(resolution = 0.4). The resolution used was determined by using the clustree package.^138^ After identifying enriched marker genes using FindAllMarkers(test.use = “roc”, only.pos = TRUE, logfc.threshold = 0.0, min.pct = 0.0), clusters were annotated. Afterward, markers were filtered using a “area under the curve” (myAUC) cutoff of >0.7. Following clusters were merged as they did not harbor individual biologically significant markers: testes related (cluster 5,4), late male germ cells (cluster 2,7,6,0). With the parameters used, the gut cluster was not automatically detected as a separate cluster and was as such manually defined using the expression of conserved markers for this tissue, which are included in Table S5.

#### Pathway analysis

Enriched Gene Ontology (GO) terms were identified using the topGO package.^117^ Terms were obtained from WormbaseParasite Version 17 and supplemented by running InterProScan v 5.60-92-0.^116^ Analysis was performed using the weight01 method for all categories (BP, MF). The node size for each category was restricted to ≥ 4 and significance was determined using Fisher’s exact test against all expressed genes. KEGG analysis was performed using the R package clusterprofiler (version 4.18.4). To this end, we assigned *Fasciola* genes a KEGG ID by running protein sequences through kofamKOALA^139^ and eggNOG-mapper.^140^ Pathway analysis was performed using a set of curated pathways in clusterProfiler^119^ (version 4.10.1). Lists of assigned GO terms and KEGG IDs as well as screened pathways are provided within the zenodo repository attached to this project.

#### STRINGdb analysis

Molecular interactions were predicted using the STRING online tool (v12)^141^ after uploading the *F*. *hepatica* proteome (PRJNA179522). The top 100 marker genes based on their AUC values (Table S2) for the cluster of interest was used as input with default settings.

#### RNA velocity

For RNA velocity analysis, loom files were created using velocyto^125^ based on alignments generated by the cellranger pipeline. The resulting data was filtered afterward to only contain information from the early and late vitelline cell clusters using barcode and cluster information extracted from Seurat. Subsequent analysis was performed in scvelo^60^ using the default parameters and the dynamical model for calculating velocities. Prior to analysis, some genes were removed due to expression pattern invalidating the model assumptions following the workflow described by Barile et al.^142^

#### Cell-cycle scoring

Assignment of cells to their corresponding cell-cycle state was done using the CellCycleScoring function in Seurat.^115^ The necessary marker genes needed for this analysis were identified by similarity to a previously published^143^ gene set for humans by BLAST using an E-value cut-off of 1×10^−5^. To this end, protein sequences were extracted from Ensembl.^144^ A list of the identified genes can be found in Table S8.

Table S8. List of identified cell-cycle marker genes by BLAST

#### Spatial integration

Mapping into the spatial transcriptome data of *F*. *hepatica* adults^26^ was achieved using the anchoring approach implemented into Seurat.^46^ Both datasets were normalized using SCTransform and anchors were identified, with the single-cell data as a reference. The TransferData function was used to calculate prediction scores, using the first 9 principle components to weight anchors.

#### Kinase sequence comparision

For identification of PAK kinases in other parasitic flatworms, the protein sequences of selected worms (*Clonorchis sinensis* - PRJNA386618, *Echinococcus granulosus* - PRJNA754835, *Echinococcus multilocularis* - PRJEB122, *Opisthorchis viverrini* - PRJNA222628, *Schistosoma haematobium* - PRJNA78265, *Schistosoma japonicum* - PRJNA520774, *Schistosoma mansoni* - PRJEA36577, *Taenia solium* - PRJNA170813) were downloaded from WBPS19. Next, we profiled the sequences for the presence of an N-terminal PDB (PF00786) and a kinase domain (PF00069) by utilizing hmmsearch implemented in the HMMER v3 software suite.^145^ Sequences with a protein kinase domain and an N-terminal PDB were considered a PAK and retained. Kinase domains were extracted using the software package seqkit.^127^ Both the sequences of domains and full-length kinase sequences were pairwise aligned using needle from the EMBOSS software suite.^126^ A pairwise identity matrix was constructed from these comparisons and visualized as a heatmap using the pheatmap package in R.^146^ Hierarchical clustering was applied using the default settings in pheatmap.

#### Phylogenetic analysis

*F*. *hepatica* PAK orthologs were identified based on the presence of PDB and kinase domains, which was additionally verified by SMART v 9.0 of amino acid sequences.^118^ The sequences of PAK family members from other selected organisms were obtained from various databases (Table S6). In order to construct the tree, the sequences were first aligned using MAFFT (version 7.505) with the L-INS-i setting.^122^ The alignment was subsequently refined using MUSCLE (version 5.1).^123^ The processed sequences were used for bayesian tree construction with MrBayes version 3.2.7a.^121^ Posterior probabilities were calculated as recommended with 1,000,000 trees sampling every 100th tree. The first 25% was discarded as burn-in and a majority rule tree was constructed. The finalized tree was drawn using the Interactive tree of life.^124^

#### *In vitro* culture and inhibitor treatment

The activity of the PAK4 kinase inhibitor LCH-774994 (Selleckchem, USA) on parasite vitality was assessed *in vitro*. Worms were cultured in either 12-well plates (for adults and immature worms) or 48-well plates (for NEJs) in RPMI1640 containing 10% chicken serum and 1% ABAM. The inhibitor was added in varying concentrations and the solvent dimethyl sulfoxide (DMSO) was used as a control. The worms were incubated at 37 °C and 5% CO_2_ over a period of 72 h, during which media and inhibitor was refreshed every 24 h. The motility of the worms was scored every 24 h using a stereomicroscope and the following scoring scale: 3 (normal motility), 2 (reduced motility), 1 (minimal and sporadic movements), and 0 (no movement even upon mechanical stimulation with tweezers).

#### *In situ* hybridization

For the generation of probes, we employed a strategy previously outlined^147^ with minor modifications. Shortly, sequences were amplified from cDNA of adult worms and cloned into the plasmid pJC53.2. For validation, all obtained clones were sequenced prior to their usage in any experiments. A list of the used primers, as well as sequencing results for the probes in use can be found in Table S7. The probes were amplified from the plasmid and the resulting PCR product was used in an *in vitro* transcription, labeling the probe with digoxigenin-12-UTP or fluorescein-12-UTP (Jena Bioscience, Germany) using either SP6 or T3 RNA polymerase (Roche, Switzerland). Synthesis was carried out overnight at 28 °C and the probes were precipitated with 7.5 M lithium chloride at −80°C for 1 h. As a control for each gene, the corresponding “sense” sequence was also synthesized as riboprobes.

Table S7. List of primers used in this study

*In situ* hybridization, either colorimetric (ISH) or fluorescent (FISH), was carried out as outlined in Cancela and Maggioli 2020^148^ with some modifications. In brief, worms were embedded flat in cryomolds (Sakura Finetek, Germany), frozen on dry ice and stored at −80°C until further use. Transversal sections were prepared with a thickness of 10 μm using a cryostat and stored at −80°C again until further use. The sections were thawed at room temperature for 30 min, then fixed with 3.7% formaldehyde in PBS, and permeabilized with PBS containing 0.3% Triton X-100. Following permeabilization, the sections were treated with 4× SSC buffer containing 0.03% hydrogen peroxide to quench residual peroxidase activity. For hybridization, the samples were first prehybridized with hybridization buffer before adding a probe in a concentration of 1 ng/μL at 55 °C. After overnight incubation, the sections were washed with hybridization wash buffer at 55 °C followed by a series of 2×, 0.2× and 0.1× SSC buffers each at 55 °C for 10 min. The samples were blocked with blocking solution for 1 h, and anti-DIG-AP (1:1000) or anti-DIG-POD (1:200, Roche, Switzerland) antibodies were added in blocking solution at room temperature for 2 h. After washing FISH samples in MAB-T buffer, development was carried out using the TSA Cyanin 3 system (Akoya Bioscience, USA) for 10–15 min. For development of a second probe residual peroxidase activity was quenched with 100 mM sodium azide. After washing with PBS the samples were incubated with anti-FITC-POD (1:200, Roche, Switzerland) in blocking solution for 2 h at room temperature. After washing again with MAB-T buffer, development was carried out using TSA Fluorescein system (Akoya Bioscience, USA). The sections were then counterstained with 1 μg/mL Hoechst 33342 and mounted in ROTImount FluoCare (Carl Roth, Germany). Colorimetric development was carried out with substrates NBT and BCIP in AP buffer until desired signal strength was reached. Samples were dehydrated in ethanol and mounted in 80% glycerol. The sense probes for each gene served as a control and are included in Figure S15 for colorimetric and Figure S16 for fluorescent development.

#### EdU staining

EdU staining in adult worms was carried out using the Click-iT EdU Cell Proliferation Kit (ThermoFisher, UK). Following a 24 h exposure with 500 μM EdU dissolved in dH_2_O, the worms were frozen flat in OCT and stored at −80°C. Sectioning was performed as described above. The Click reaction was performed following the manufacturers’ description after completion of *in situ* hybridization. Afterward, the sections were washed and counterstained using 1 μg/mL Hoechst 33342.

#### Immunostaining

Immunostaining was applied on cryosections after completion of RNA *in situ* hybridization. The sections were washed in PBS and afterward blocked for 1 h in blocking buffer (5% goat serum in PBS with 0.5% Tween 20). Incubation with the primary antibody (6G10, Developmental Studies Hybridoma Bank deposited by Zayas R.M or 3C11, Developmental Studies Hybridoma Bank deposited by Buchner E) was performed overnight in a dilution of 1:200 in blocking buffer. The next day, the sections were washed in PBS with 0.5% Tween 20 and incubated for 2 h with the secondary antibody (goat anti-mouse IgG, ThermoFisher). Finally, the sections were counterstained using 1 μg/mL Hoechst 33342 and mounted in ROTImount FluoCare.

#### Microscopy

For microscopy, a Leica DM IL inverted microscope was used for chromogenic ISH pictures. Fluorescent signal was detected using either a TCS SP5 vis confocal laser scanning microscope (CLSM; Leica Microsystems, Germany) or a Olympus IX81 fluorescent microscope (Olympus, Japan). Alexa Fluor 488 was excited using an argon-ion laser at 488 nm, and Hoechst 33342 at 405 nm. Cyanin 3 was excited at 514 nm argon-ion laser.

#### Molecular docking

We performed molecular docking to investigate the binding of the selected inhibitor to the *Fasciola* PAK4. A homology model was generated using the SWISS-MODEL webserver,^128^ based on the human PAK4 structure (RCSB PDB ID: 2CDZ), which had a 61% sequence identity. Additionally, we utilized a predicted 3D structure of FhPAK4, which was available from the AlphaFold database (UniProt: A0A4E0RB72; AlphaFold: AF-A0A4E0RB72-F1-v4).^129^^,^^149^^,^^150^ The center coordinates and dimensions of the docking box were calculated by aligning the human PAK4 structure onto FhPAK4. This involved aligning the crystal structures of human PAK4 onto FhPAK4 to ensure that it was appositely covered. LCH-7749944 was prepared from SMILES and converted to mol2 format with protonation corresponding to ph 7.4 using the TLDR webserver (https://tldr.docking.org/).^130^ Another mol2 file for the LCH-7749944 ligand was generated via OpenBabel v3-1-1-1.^131^ The mol2 format was further minimized for the docking studies using the MMFF94^151^ force field parameter. For docking studies, the receptor and the ligand were prepared using the AutoDock scripts (prepare_ligand.py and prepare_receptor.py).^132^^,^^152^ Docking studies were performed using AutoDock Vina v1.1.2 with an exhaustiveness parameter set to 64. Docking was performed a total of four times, using combinations of the predicted structures (AlphaFold and SWISS-MODEL) and both ligand versions respectively. This yielded 20 docked conformations of the ligand per docking round. Steepest descent energy minimization was executed on the poses via UCSF ChimeraX.^133^ The best ligand pose from all four docking rounds was visualized using PyMOL^153^ for 3D and PoseEdit/PoseView for 2D visualization using the ProteinsPlus webserver.^134^^,^^154^^,^^155^

### Quantification and statistical analysis

Statistical significance of measured effects on inhibitor-treated parasites was determined using the Mann-Whitney U pairwise rank-sum test implemented in R with Holm correction to control for multiple testing. Information on the number of biological replicates can be found in the respective figure legend.

## Supplemental information

Document S1. Figures S1–S16

Table S1. List of samples used and related QC metrics

Table S2. List of marker genes per cluster

Table S3. GO terms enriched in clusters

Table S4. KEGG pathways enriched per clusters

Table S5. List of cathepsins used for definition of the gut cluster

Table S6. Sequences and accession numbers used for phylogenetic analysis of PAK
