# Supplementary material for: Single-cell transcriptomics identifies a p21-activated kinase important for survival of the zoonotic parasite Fasciola hepatica
Source: iScience. 2026 Jul 16;29(8):116778. doi: 10.1016/j.isci.2026.116778 (PMC13400776; doi:10.1016/j.isci.2026.116778)
Supplement: Document S1. Figures S1–S16 [file mmc1.pdf]

iScience, Volume *29*

Supplemental information

Single-cell transcriptomics identifies a p21-activated kinase important for survival of the zoonotic parasite *Fasciola hepatica*

Oliver Puckelwaldt, Svenja Gramberg, Sagar Ajmera, Janine Koepke, Jamal Shamsara, Christos Samakovlis, Peter Kolb, and Simone Haeberlein

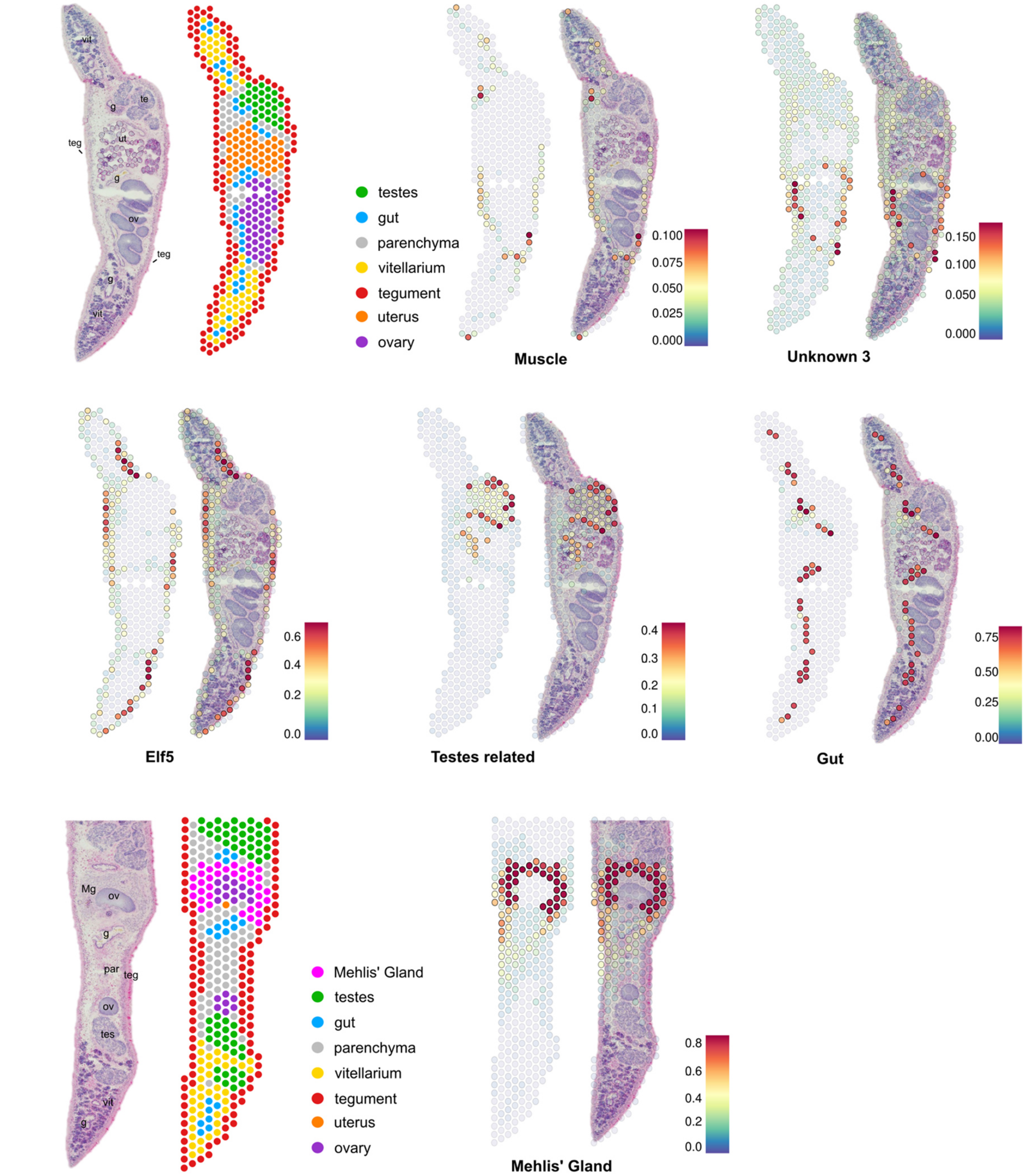

Fig S1 Confirmation of cluster annotation by integration into spatial transcriptome data. H&E stained section and spatial projection of spots covering the tissue modified after Gramberg et al 2024. The color of the spots corresponds to the associated prediction scores for the clusters.

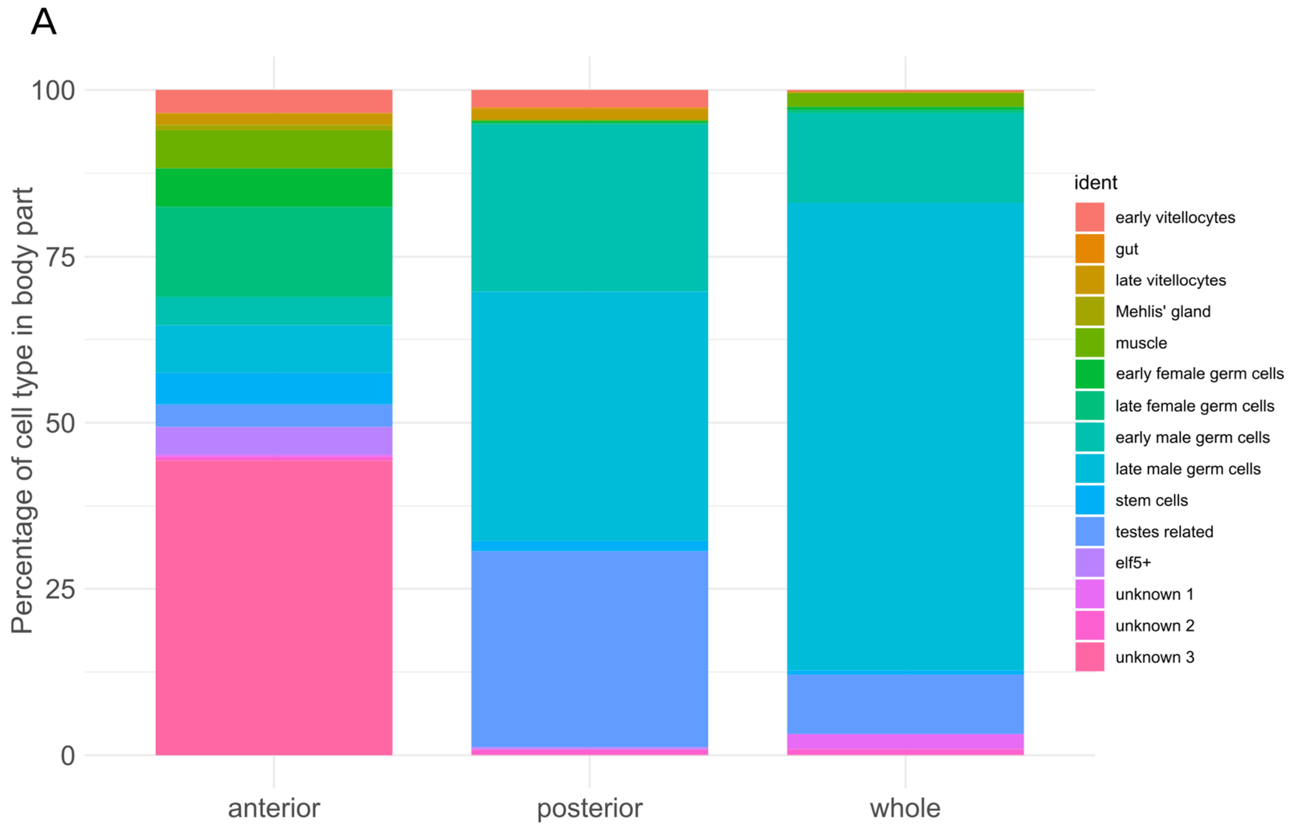

Fig S2 Cellular composition differs between samples. For each sample type either anterior, posterior or whole worm sample, the number of cells within the clusters was computed. Shown is the percentage composition of each cluster per sample type.

A

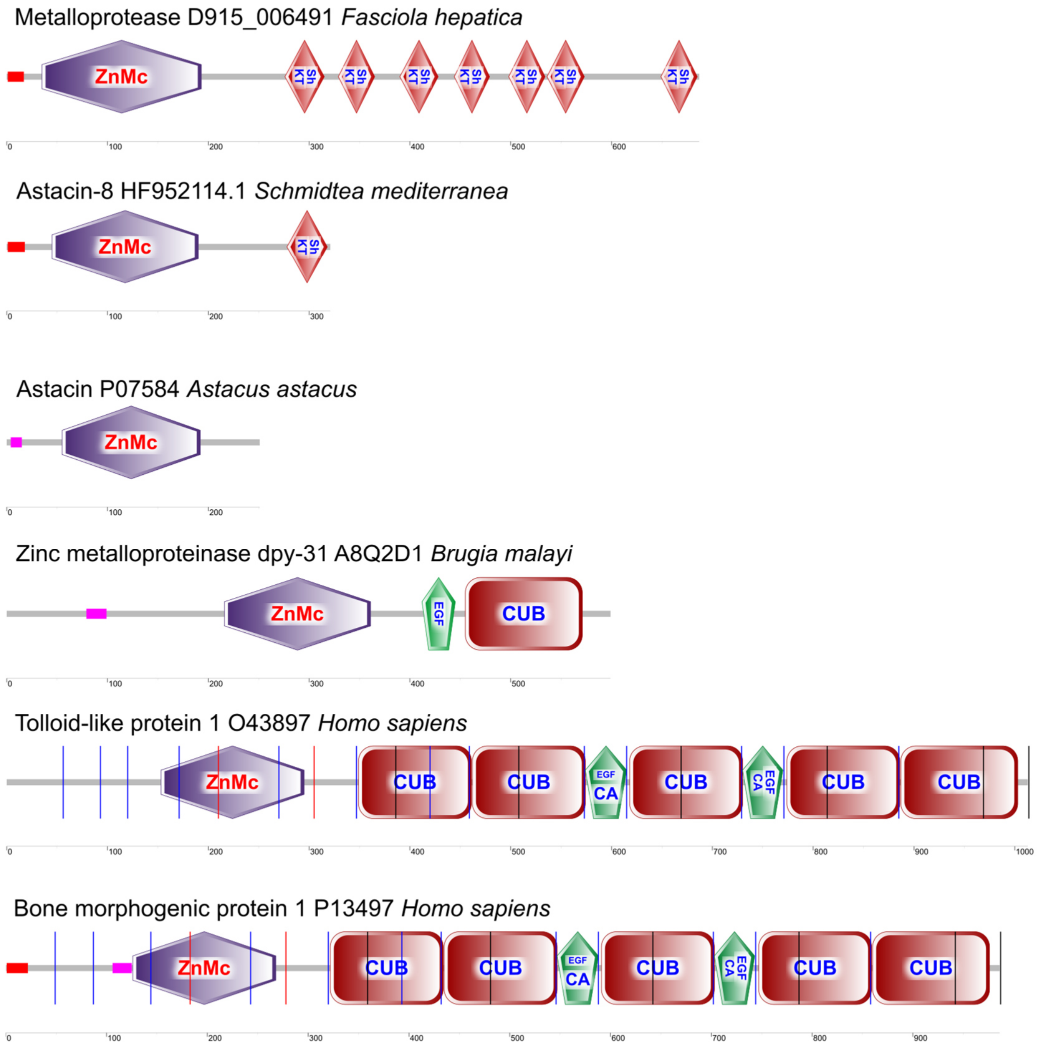

B

Astacin Zinc binding domain

HEXXHXXGFXHXXRXDR

```
D915_006491 Fasciola hepatica      GRNEKYCHAQLGVVN-DGRHELILSPLCRSFGQILHELTHALGLMHELMRPDRDDYIIVH      100
HF952114    Schmidtea mediterranea RRGSGGCNAILGQDRKKGTQTVNLGIGCRWYGLFLHELGHTIGLHHEHQHPDRDIGVSVQ      112
P07584 Astacus astacus             FTDVDGCASNVGRHPLGEPQFVSLAPECIRLGVIAHEVAHALGFWHEQSRPDRDNYVTVR      111
A8Q2D1 Brugia malayi               FTSGSGCWSYVGRIS-GAQQVSLQANGCVYHGTIIHELMHAIGFYHEHTRMDRDNYVTIN      111
O43897 Homo sapiens                TYRPCGCCSYVGRRG-NGPQAISIGKNCDKFGIVVHELGHVIGFWHEHTRPDRDNHVTII      108
P13497 Homo sapiens                TYRPCGCCSYVGRRG-GGPQAISIGKNCDKFGIVVHELGHVVGFWHEHTRPDRDRHVSIV      108
                                         * : :*       :    .  *   * . **: *.:*: **  : ***  : :
```

Fig S3 Identification of an astacin-like protease in the *F. hepatica* genome. A SMART analysis of the liver fluke metalloprotease and selected astacin sequences. **B** Alignment of the zinc-binding domain of selected sequences.

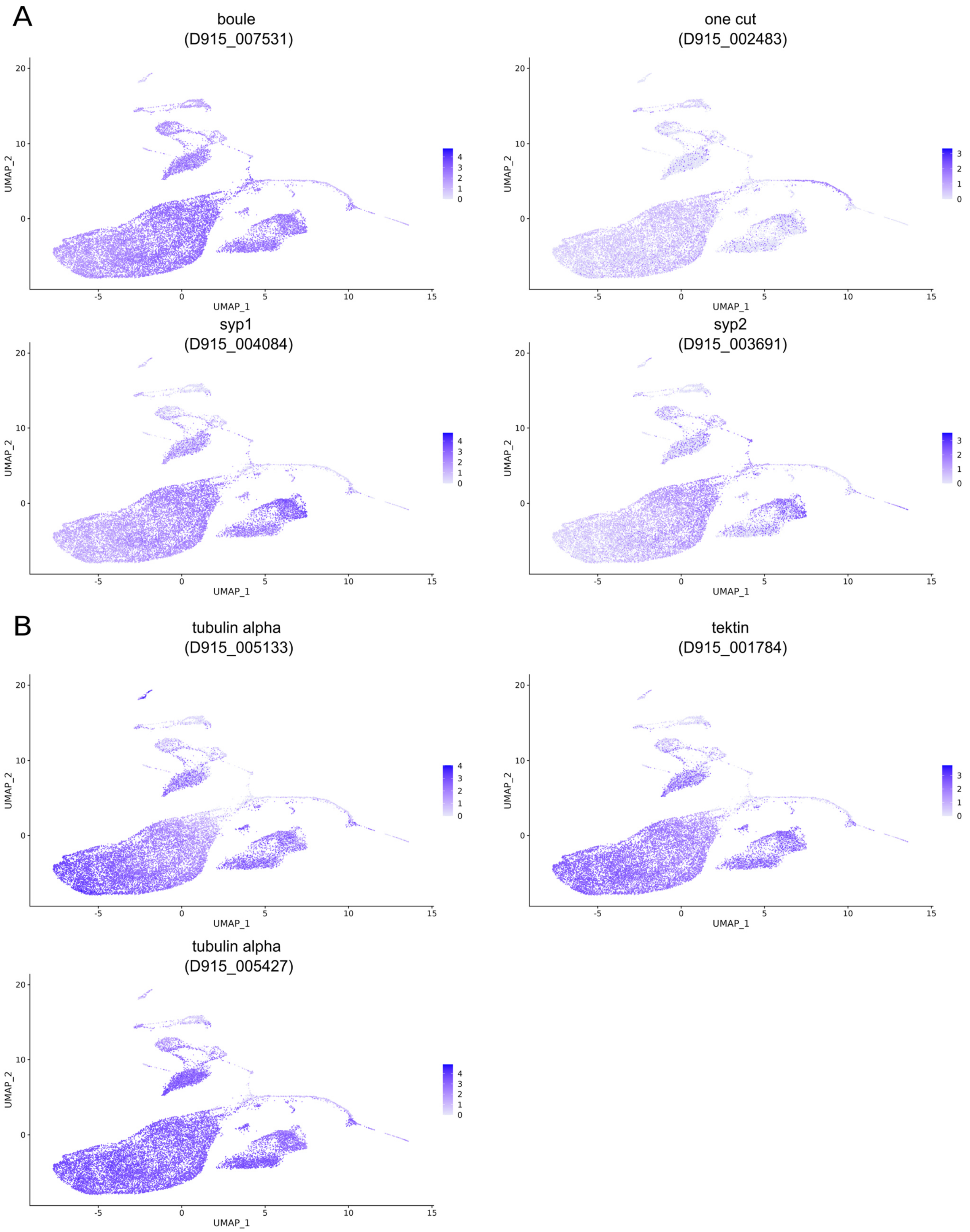

Fig S4 Expression of selected marker genes in spermatocyte clusters. UMAP plots colored by gene expression showing the expression of selected marker genes for **A** Cluster early male germ cells **B** Cluster late male germ cells.

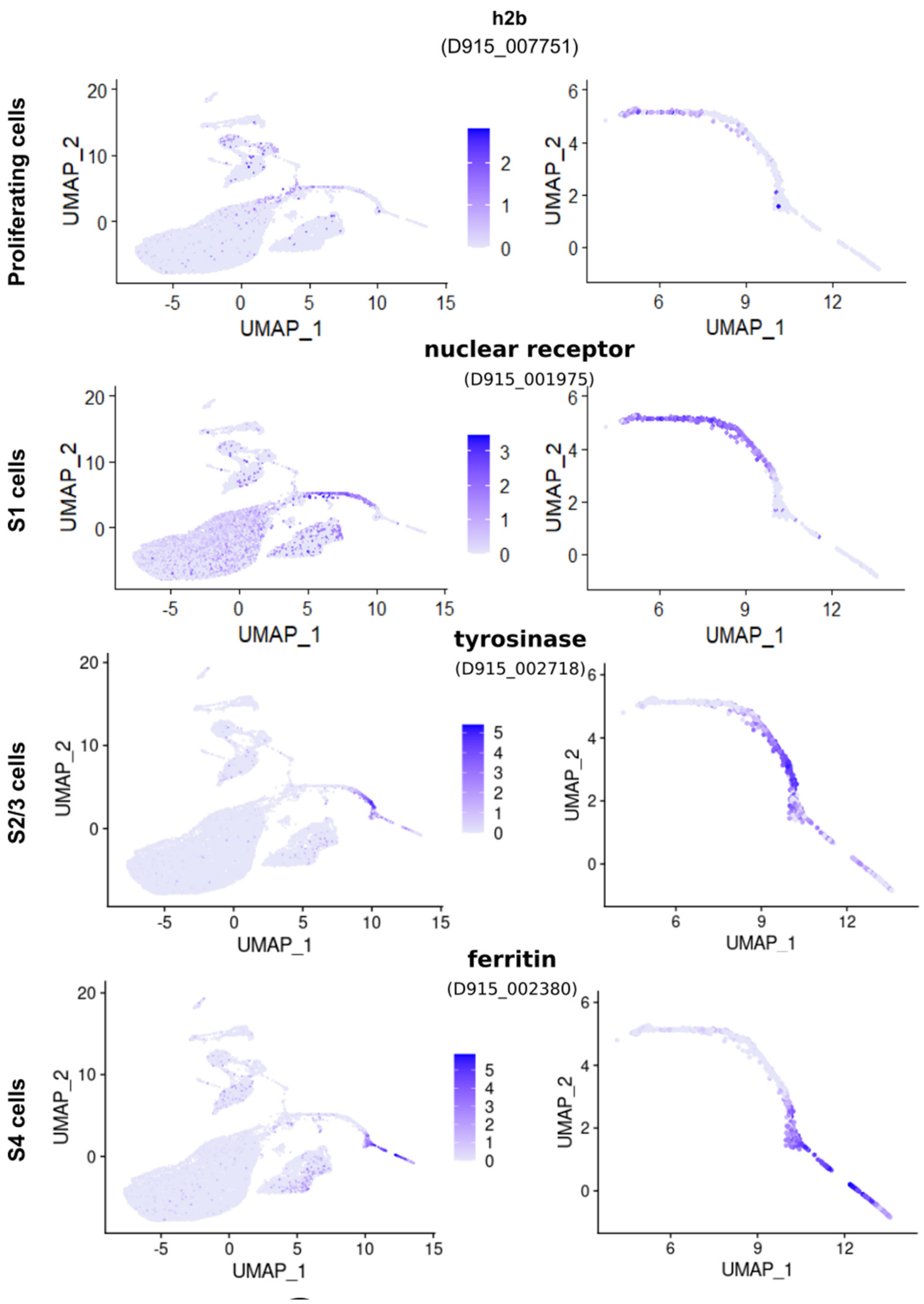

Fig S5 Genes of the vitelline lineage show distinct expression patterns. UMAP plots showing the expression of conserved vitelline-cell marker genes shared with vitelline-cell markers of *Schistosoma mansoni*. An overview of all clusters and close-up of the stem cell, early and late vitellocytes clusters is shown.

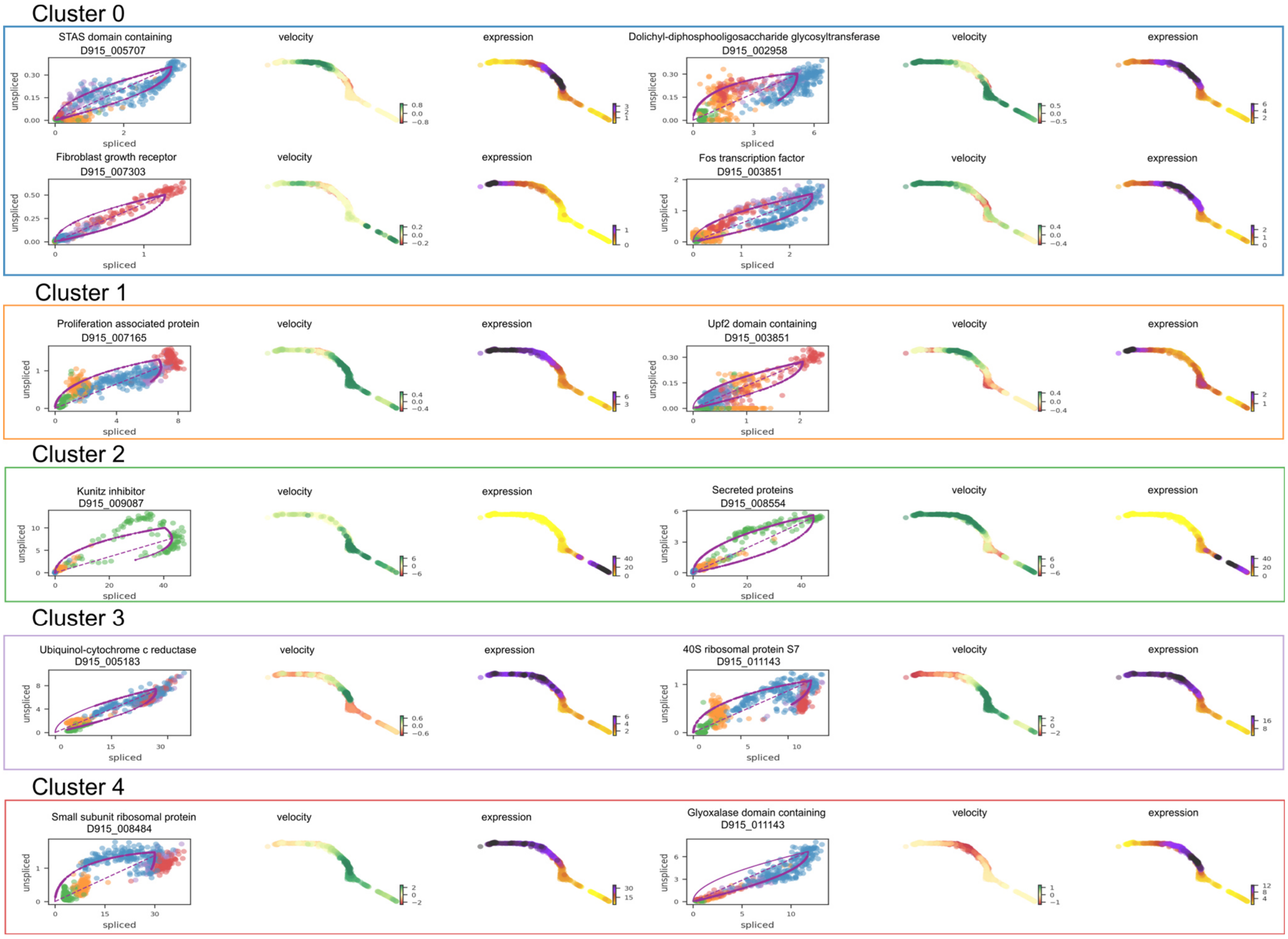

Fig S6 RNA velocity analysis of vitelline lineage reveals genes with interesting dynamics. Phase plot, velocity and expression for selected dynamical genes by cluster.

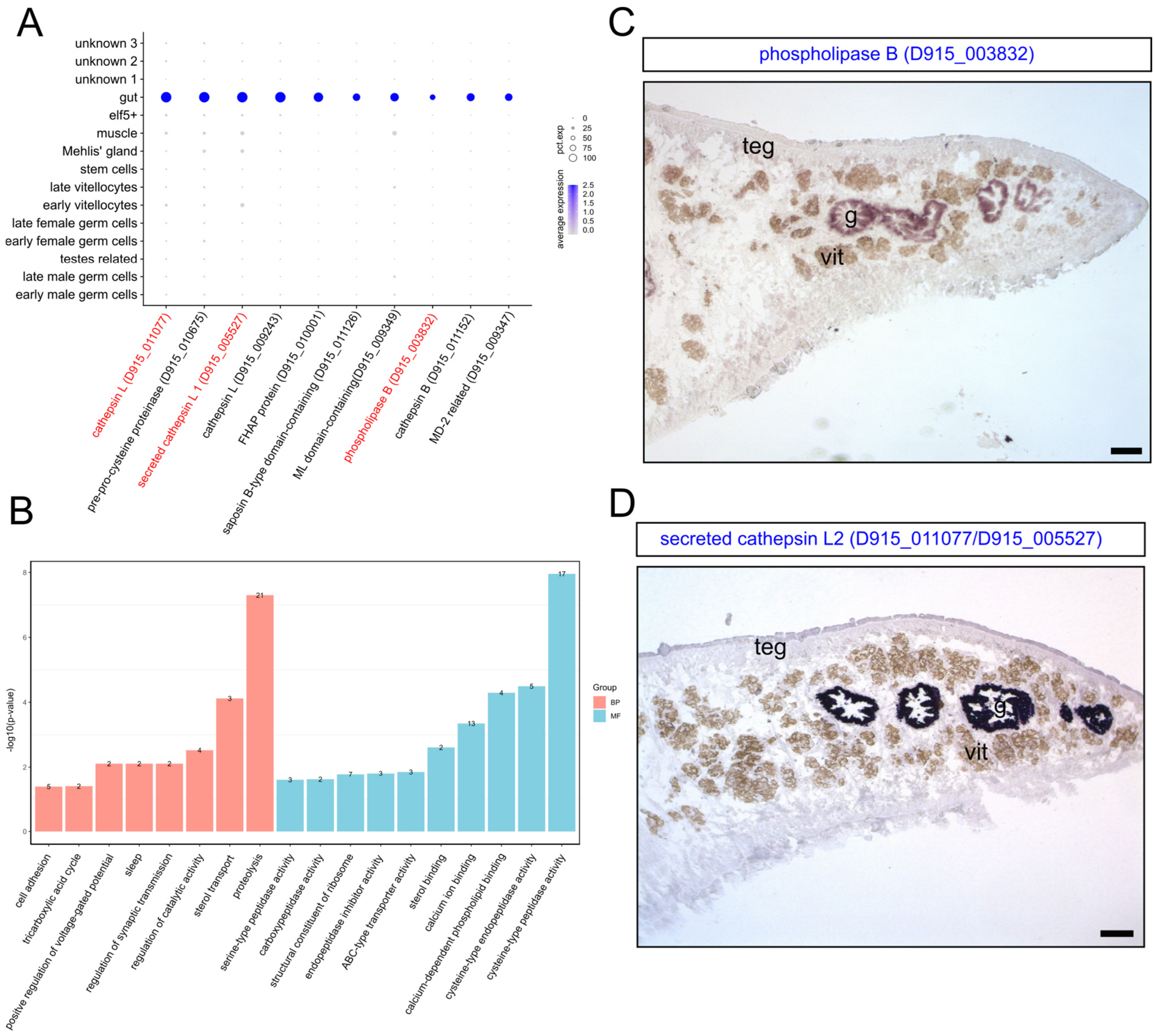

Fig S7 Gut cells of *F. hepatica* express genes involved in lipid metabolism. A Dotplot showing the expression of gut cell marker genes. ISH-validated genes are marked in red. Level of expression is indicated by color from blue (high expression) to lavender (low expression). The percentage with which cells of a cluster express the given gene is represented by the sizes of the circles. **B** Gene ontology analysis of marker genes (top 75% per cluster) revealed characteristic biological processes (BP) and molecular functions (MF). The number of enriched genes is noted at the end of each bar. **C** ISH staining for *phospholipase B* transcripts and **D** for *cathepsin L2* transcripts in the gastrodermis (blue to dark blue color). Scale bar: 100 µm. Legend: gastrodermis (**g**), tegument (**teg**), vitellarium (**vit**).

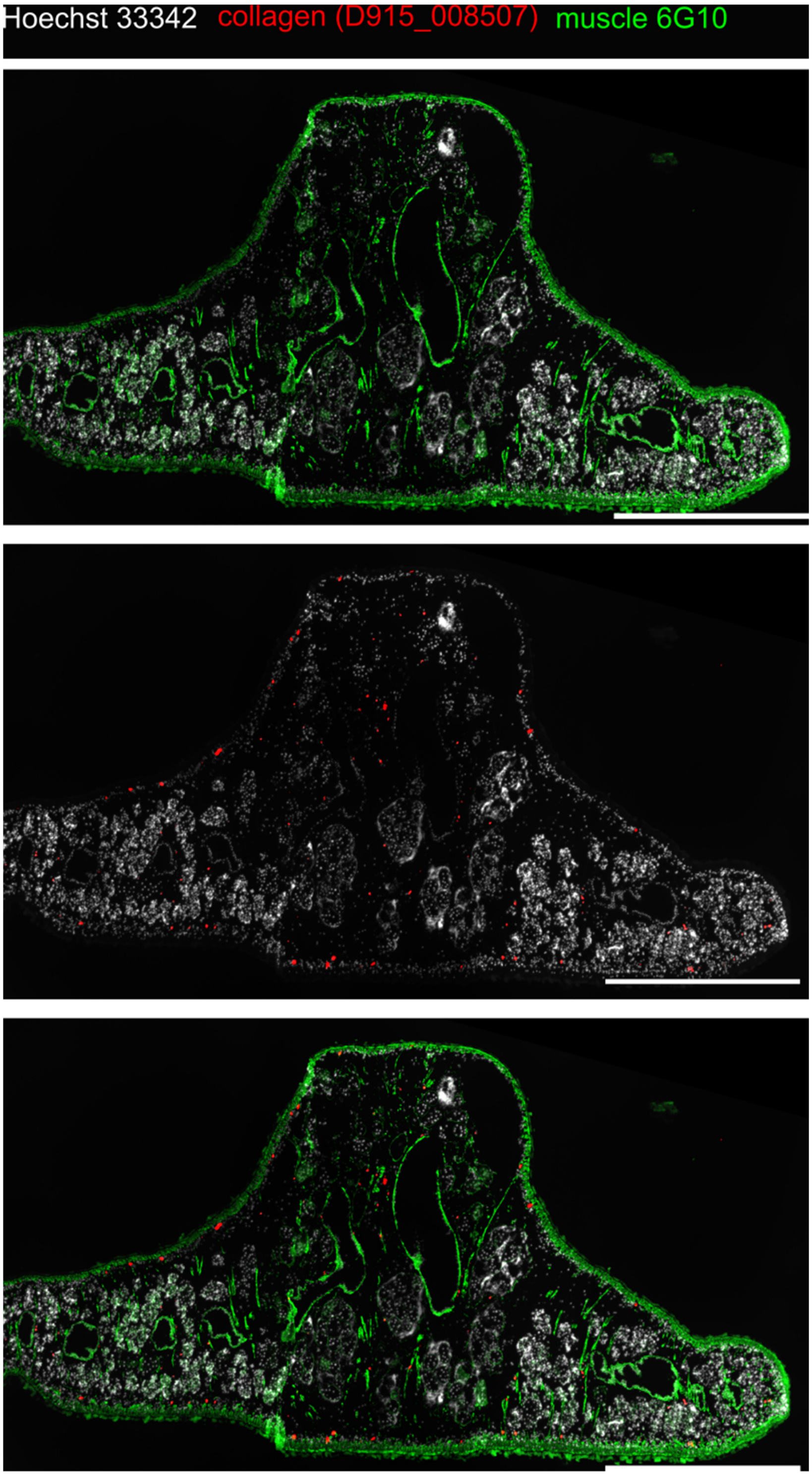

Fig S8 Muscle fibers and cells positive for collagen transcript. Transversal section co-stained for collagen transcripts by FISH (red) and muscle fiber proteins (green) by immunolocalization. Scale bar: 1000 µm.

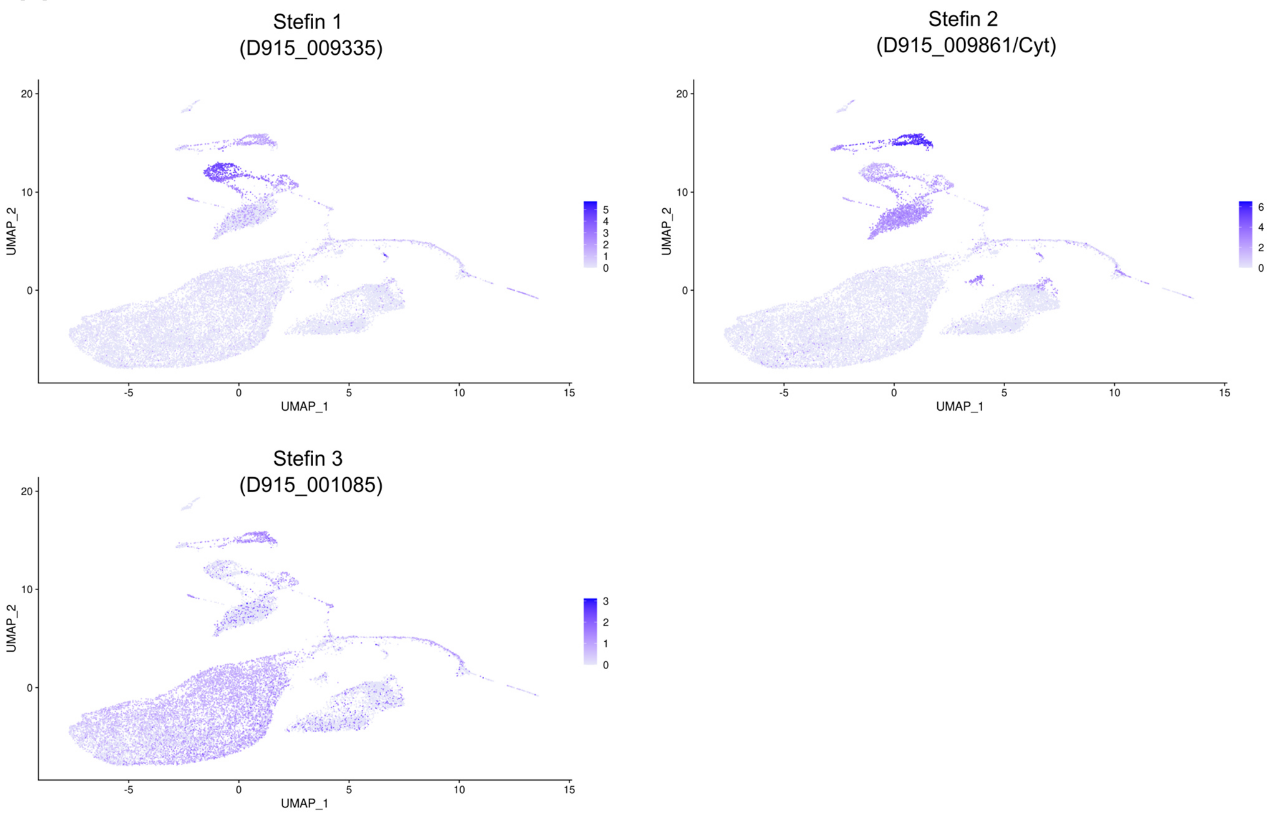

Fig S9 Different stefins show distinct expression patterns in different cell types. UMAP plots colored by gene expression showing the expression of three different stefins.

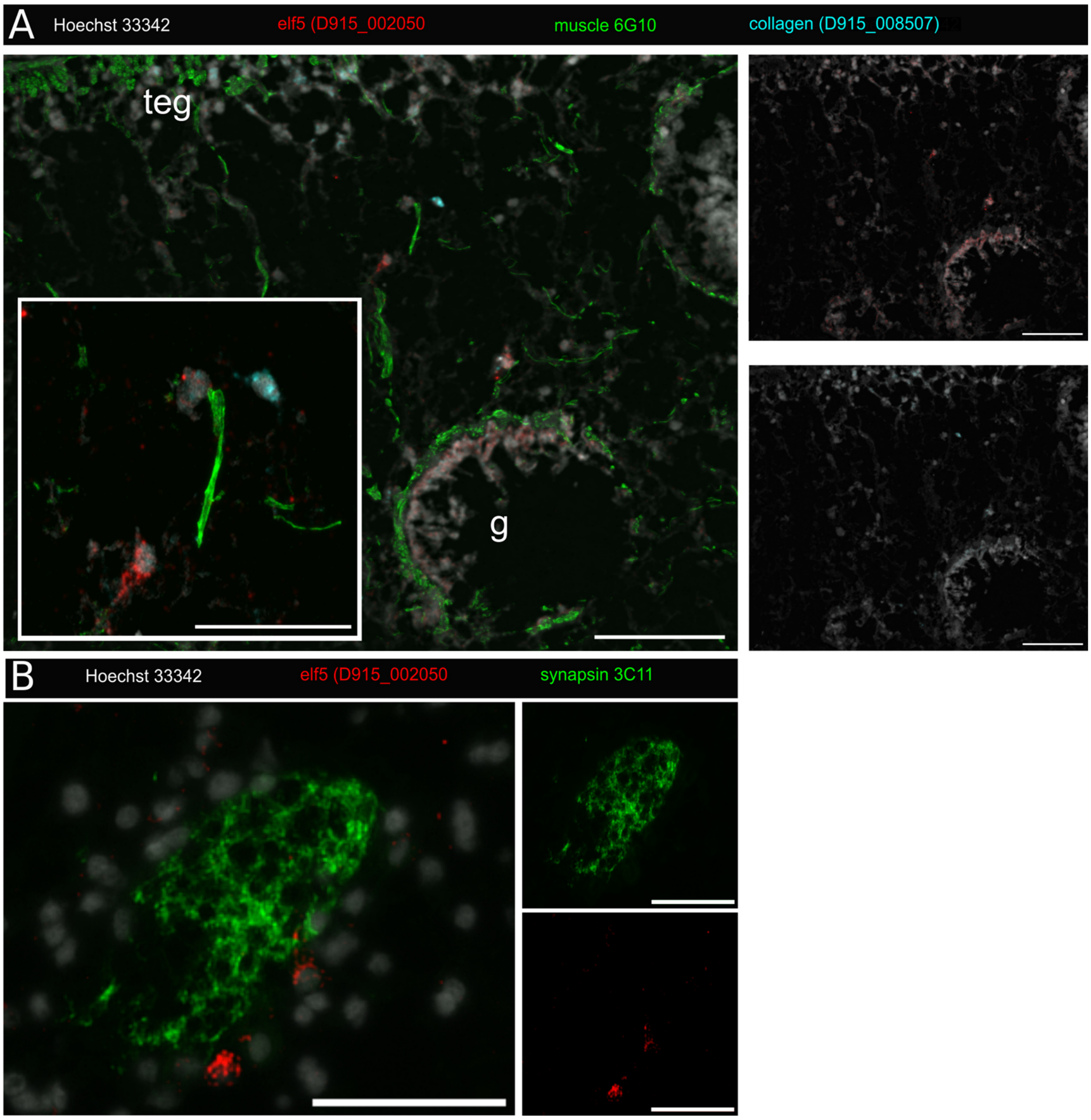

Fig S10 Elf5+ cells localize close to muscles and neurons, but are distinct cells. A FISH for *elf5* and *collagen* transcripts combined with immunolocalization of muscle fiber proteins. Scale bar: 100 µm, insert scale bar 50 µm. **B** FISH for *elf5* transcript combined with immunolocalization of synapses. Scale bar: 100 µm.

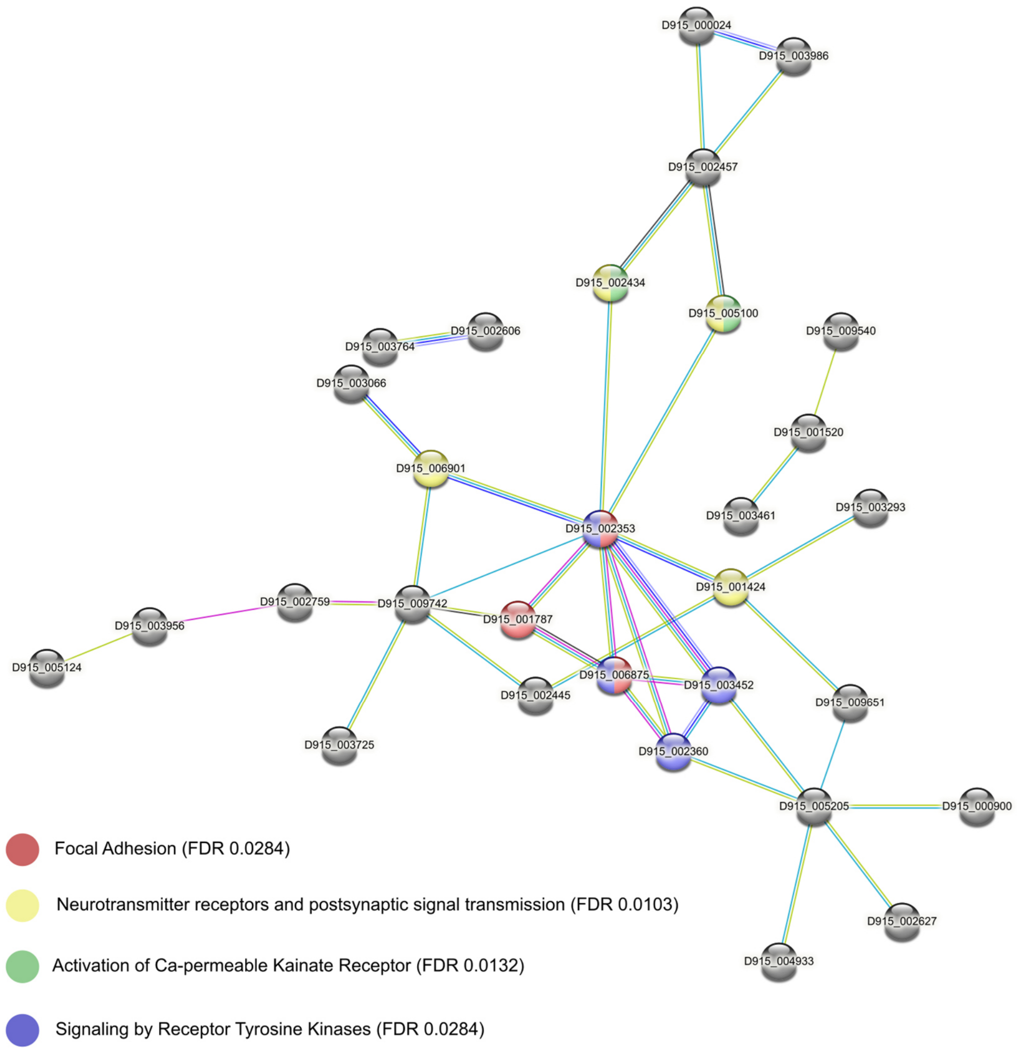

Fig S11 Identified markers show enrichment for focal adhesion network. STRING analysis of the top 100 marker genes for the elf5+ cluster connecting nodes are based on evidence of the function of homologs. Functional enrichment (*FDR*) provided by STRING.

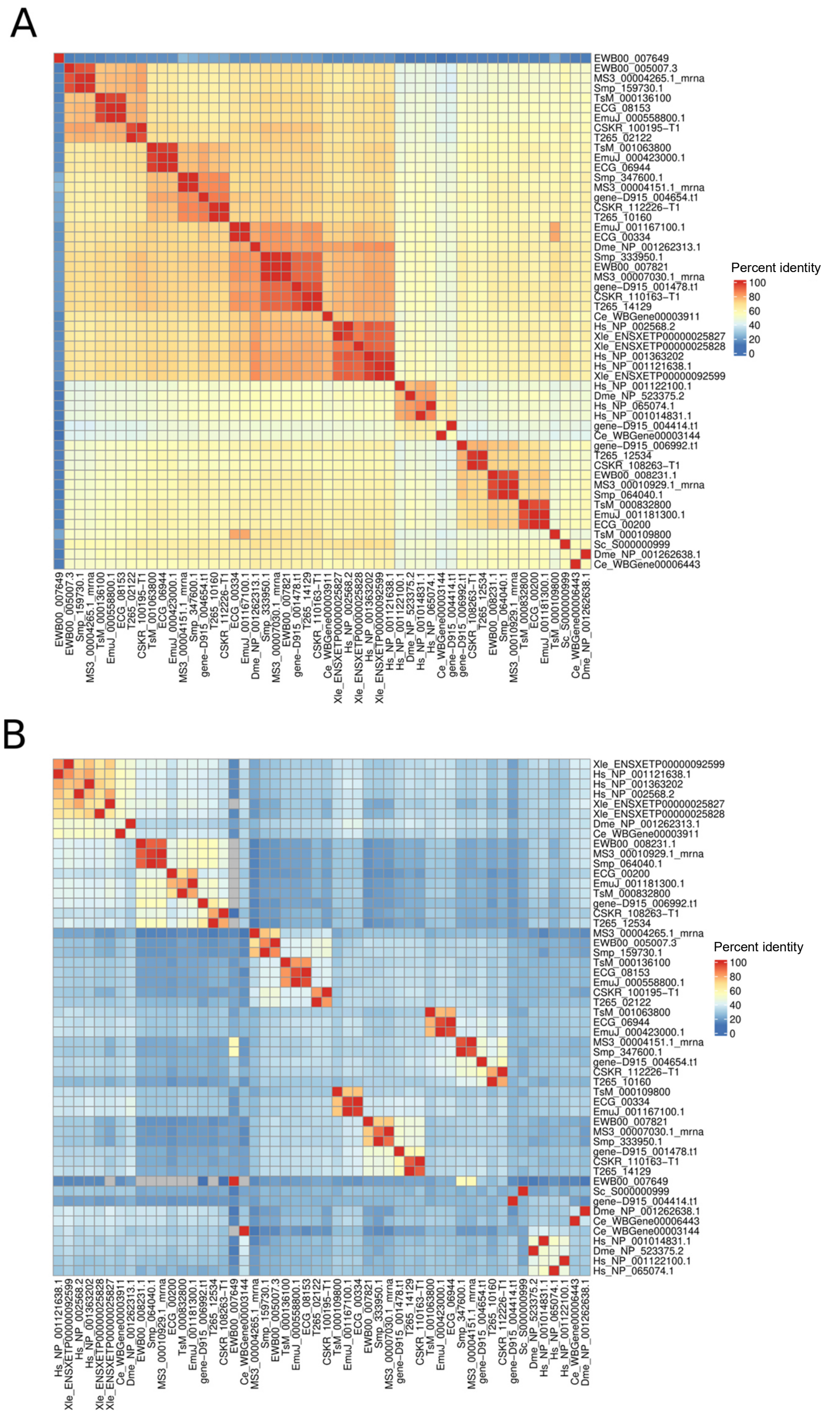

Fig S12 Pairwise identity of PAK kinases across parasitic species. Pairwise identities were calculated using kinase domain (**A**) or full length sequences (**B**) as input for pairwise alignments. Species can be identified based ID prefix: Xle – *Xenopus laevis;* Hs - *Homo sapiens;* Dme - *Drosophila melanogaster;* Ce - *Caenorhabditis elegans;* Sc - *Saccharomyces cerevisiae;* MS3 – *Schistosoma heamatobium;* TsM - *Opisthorchis viverrini;* Smp - *Schistosoma mansoni;* ECG - *Echinococcus granulosus*; EmuJ - *Echinococcus multilocularis*; CSRK - *Clonorchis sinensis*; EWB00 - *Schistosoma japonicum*; T265 - *Taenia solium*; D915 – *Fasciola hepatica.*

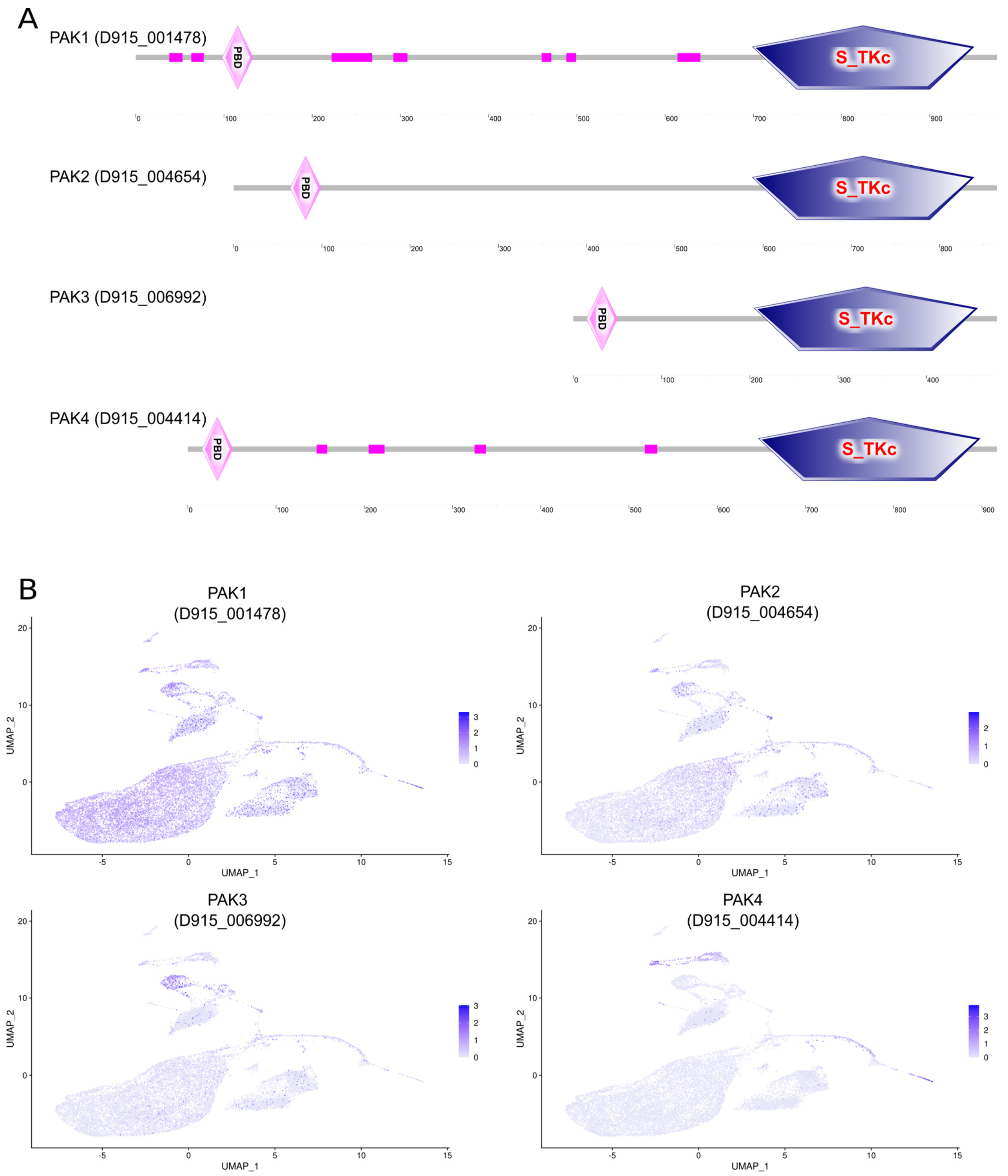

Fig S13 Domain structure and gene expression patterns of the four different PAKs. A SMART analysis confirmed the typical domain structure of PAK proteins, consisting of a serine/threonine kinase domain and a p21-binding domain. **B** UMAP plots colored by gene expression showing the expression of various *pak* genes.

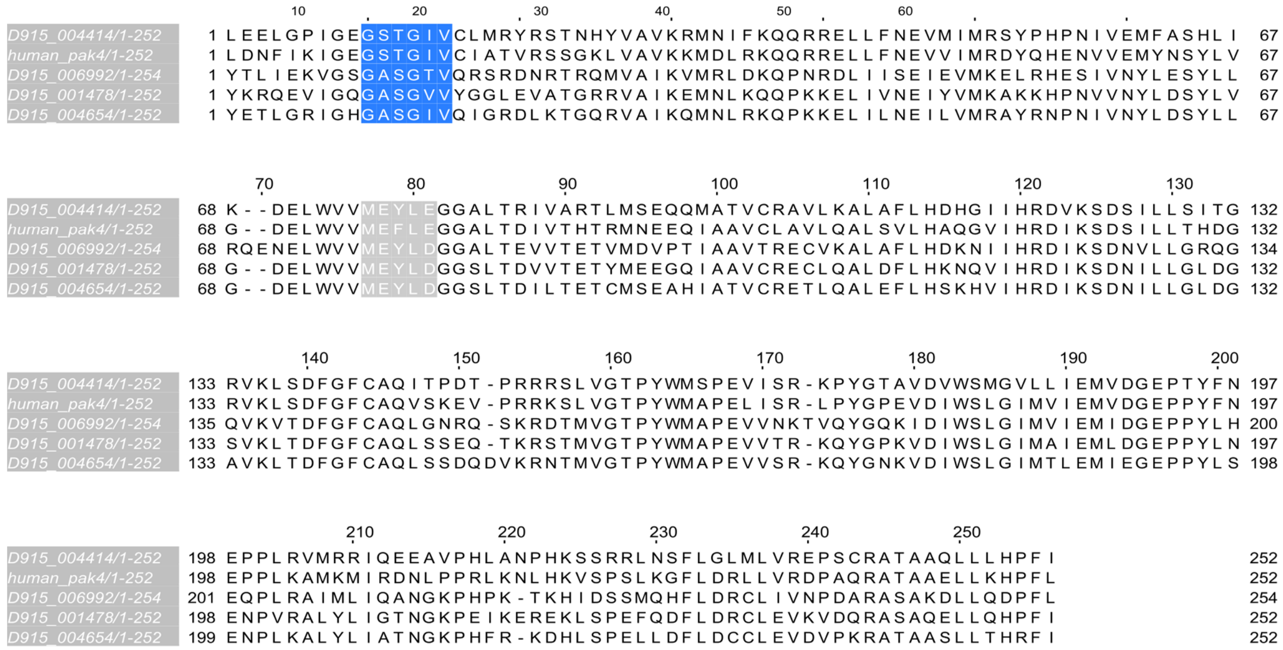

Fig S14 Alignment of the human PAK4 amino acid sequence with *F. hepatica* PAK sequences. Binding sites of LCH-7749944 are colored: P-loop in blue and hinge region in gray.

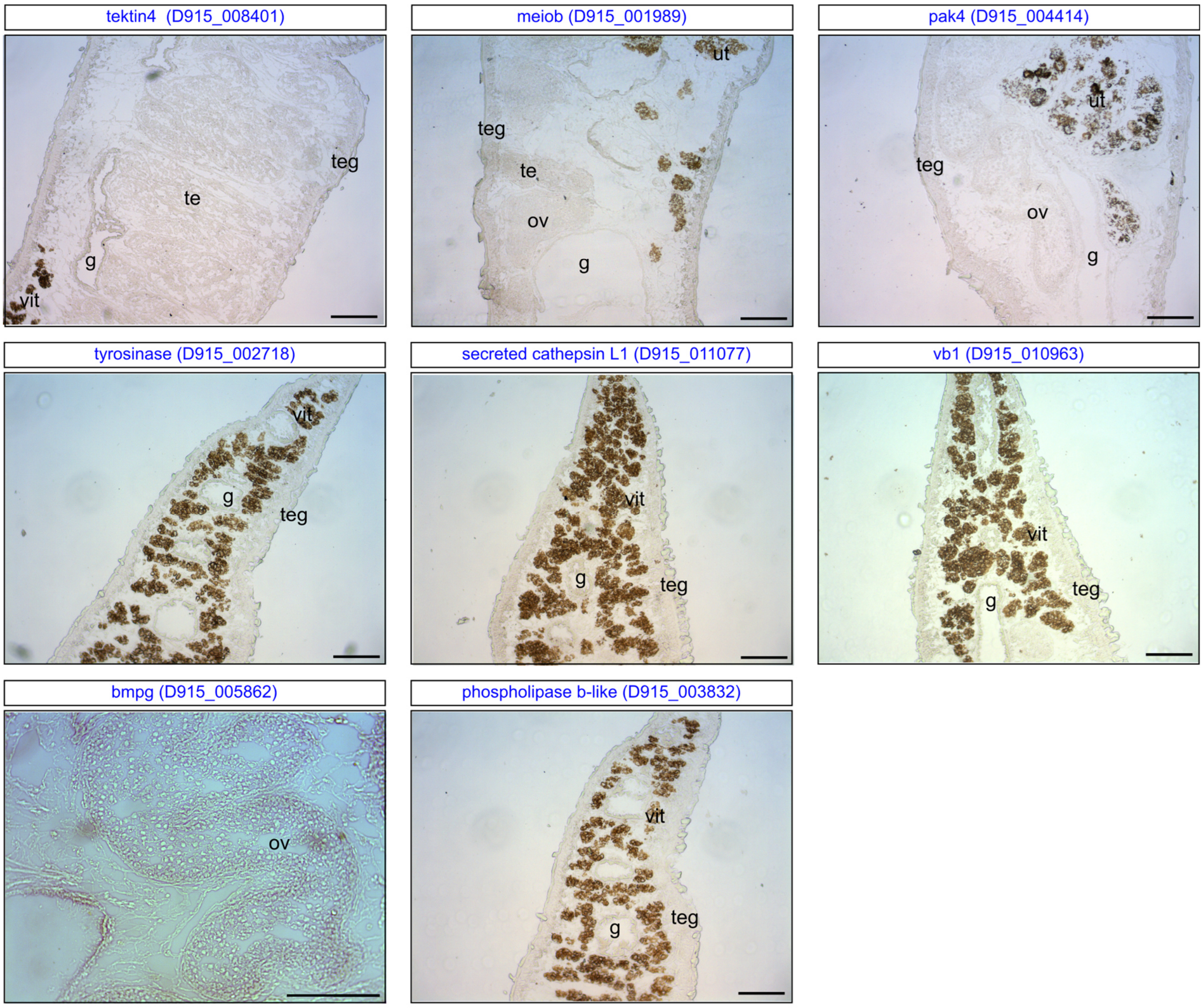

Fig S15 Sense probes produce no signal in colorimetric RNA *in situ* hybridization. Transversal section treated with sense probes as negative controls in CISH. Scale bar: 200 µm. Legend: gastrodermis (**g**), tegument (**teg**), testes (**te**), ovary (**ov**), vitellarium (**vit**).

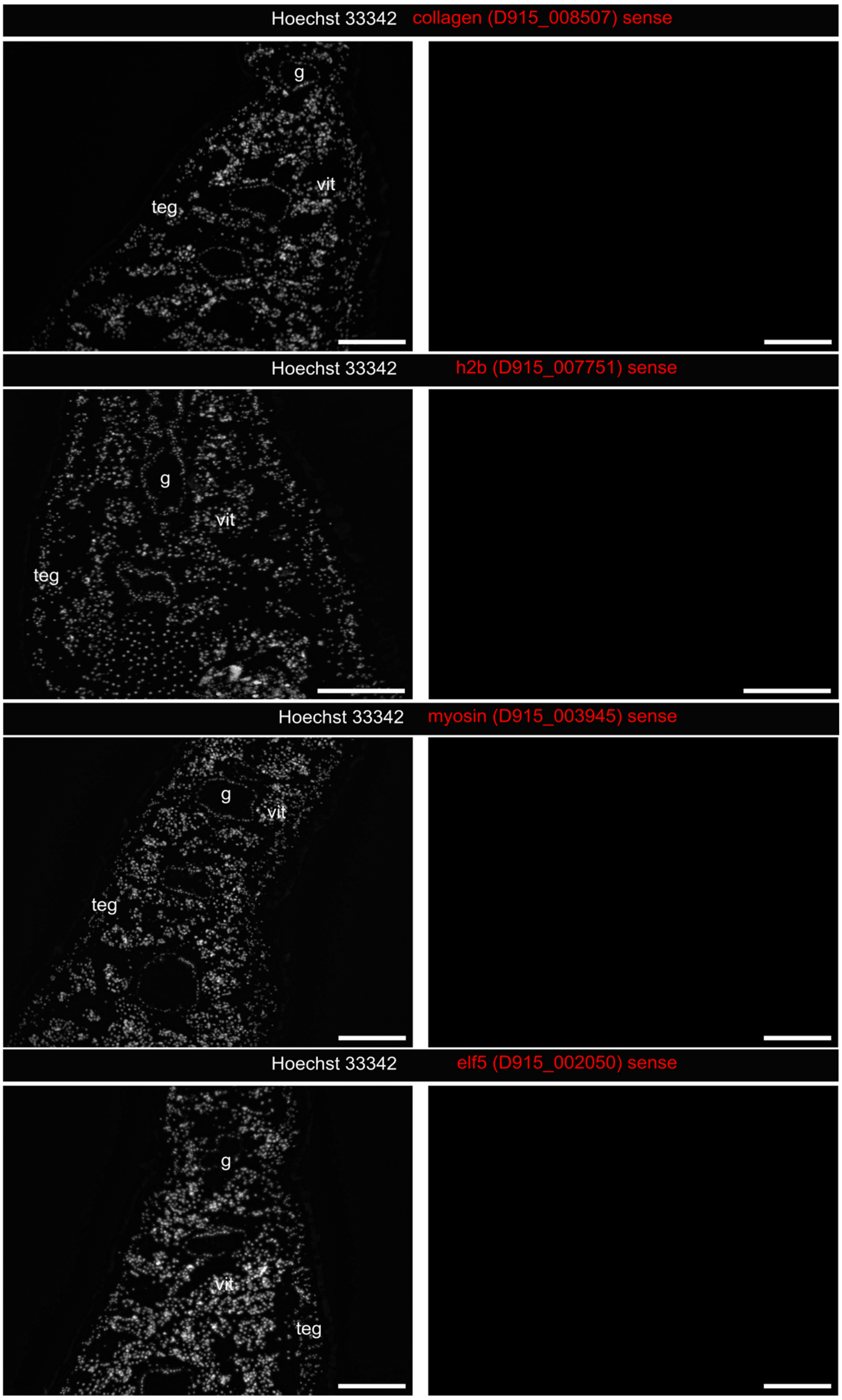

Fig S16 Sense probes produce no signal in fluorescent RNA *in situ* hybridization. Transversal section treated with sense probes as negative controls in FISH. Scale bar: 200 µm. Legend: gastrodermis (**g**), tegument (**teg**), vitellarium (**vit**).
